# Computer Vision-Based Fire–Ice Ion Algorithm for Rapid and Nondestructive Authentication of Ziziphi Spinosae Semen and Its Counterfeits

**DOI:** 10.3390/foods14010005

**Published:** 2024-12-24

**Authors:** Peng Chen, Xutong Shao, Guangyu Wen, Yaowu Song, Rao Fu, Xiaoyan Xiao, Tulin Lu, Peina Zhou, Qiaosheng Guo, Hongzhuan Shi, Chenghao Fei

**Affiliations:** 1 Institute of Chinese Medicinal Materials, Nanjing Agricultural University, Nanjing 210095, China; chenpenggzy@163.com (P.C.); sxx@stu.njau.edu.cn (X.S.); wenguangyu@stu.njau.edu.cn (G.W.); syw@stu.njau.edu.cn (Y.S.); gqs@njau.edu.cn (Q.G.); 2 College of Pharmacy, Nanjing University of Chinese Medicine, Nanjing 210023, China; 20220675@njucm.edu.cn (R.F.); ltl2021@njucm.edu.cn (T.L.); 3 Suzhou Liliangji Health Industry Co., Ltd., Suzhou 215000, China; xiaoxyan2021@163.com; 4 Institute of Plant Resources and Chemistry, Nanjing Research Institute for Comprehensive Utilization of Wild Plants, Nanjing 210042, China; zhoupeina@163.com

**Keywords:** Ziziphi Spinosae Semen, nondestructive and fast judgment, fire–ice ion dimensionality reduction algorithm, multivariate statistics, traceability

## Abstract

The authentication of Ziziphi Spinosae Semen (ZSS), Ziziphi Mauritianae Semen (ZMS), and Hovenia Acerba Semen (HAS) has become challenging. The chromatic and textural properties of ZSS, ZMS, and HAS are analyzed in this study. Color features were extracted via RGB, CIELAB, and HSI spaces, whereas texture information was analyzed via the gray-level co-occurrence matrix (GLCM) and Law’s texture feature analysis. The results revealed significant differences in color and texture among the samples. The fire–ice ion dimensionality reduction algorithm effectively fuses these features, enhancing their differentiation ability. Principal component analysis (PCA) and partial least squares discriminant analysis (PLS-DA) confirmed the algorithm’s effectiveness, with variable importance in projection analysis (VIP analysis) (VIP > 1, *p* < 0.05) highlighting significant differences, particularly for the fire value, which is a key factor. To further validate the reliability of the algorithm, Back Propagation Neural Network (BP), Support Vector Machine (SVM), Deep Belief Network (DBN), and Random Forest (RF) were used for reverse validation, and the accuracy of the training set and test set reached 98.83–100% and 95.89–99.32%, respectively. The method provides a simple, low-cost, and high-precision tool for the fast and nondestructive detection of food authenticity.

## 1. Introduction

*Ziziphus jujube* Mill. var. *spinosa* (Bunge) Hu ex H. F. Chou (ZJS) is a thorny deciduous plant widely distributed in China [1]. Ziziphi Spinosae Semen (ZSS) refers to the dried, mature seeds of ZJS, which are derived mainly from wild sources [2]. As a traditional edible herb, ZSS was first recorded in Shen Nong’s Herbal Classic (the earliest medical monograph in China). It is classified as a top-quality herb with a variety of effects, such as being sedative and hypnotic, tranquilizing the heart, and calming the mind [3]. Modern pharmacological studies have shown that ZSS has hypnotic and sedative [4], antidepressant [5], anxiolytic [6], and antihyperlipidemic effects [7]. In addition to its application in the pharmaceutical industry, ZSS has also been developed as an additive in health food products and has received increasing attention from the food industry [8]. In recent years, the widespread use of ZSS in healthcare has led to a revisiting of its resources [9].

ZJS is mostly distributed in northern China, such as Hebei, Shanxi, Shaanxi, and Liaoning, and is mainly a wild resource [10]. In recent years, the wild germplasm resources of ZJS have not been effectively managed [11]. As a result of the “green rush” and destructive early harvesting practices, there has been a serious shortage of wild resources [12]. As one of the commonly used traditional Chinese medicines for the treatment of insomnia, with the increasing number of insomniacs, the price of ZSS has been continuously rising in recent years. This has led to the appearance of some fakes with similar characteristics and compositions, such as Ziziphus Mauritianus Semen (ZMS) and Hovenia Acerba Semen (HAS) [13]. This adulteration not only disrupts the market order but also harms consumers. Moreover, people’s increasing awareness of health issues and growing concerns about adulteration, germplasm mixing, and origin fraud suggest that food traceability is likely to become a prominent aspect of food in the future [14].

Although traditional food quality testing methods are highly accurate and reliable, they have limitations such as being time-consuming, high cost, and limited application. In addition, the inability to easily move equipment and instruments limits the application of traditional methods to meet the demand for ready-to-use inspection [15]. To address these issues, the field of food science has begun to seek faster, more convenient, and low-cost inspection techniques, and the development of computer vision (CV) and machine learning (ML) has revolutionized food quality assessment [16]. By combining computer science with robust datasets, intelligent algorithms can effectively solve food inspection problems. For example, the combination of ML and CV techniques has been used to screen apples for pests and diseases [17] or to estimate the moisture content of strawberry fruits to determine ripeness via Support Vector Machines (SVMs) and CV methods [18]. Additionally, these techniques can be used for food grading and prediction, such as identifying the banana ripening process via Xbee-Based WSN Architecture [19] or predicting the high pH thresholding of beef via near-infrared hyperspectral imaging and SVM [20]. Thus, the introduction of intelligent technologies has promising applications in the modern food industry to significantly improve product quality.

In this study, chromaticity (RGB, L*a*b*, and HSI (hue, saturation, and intensity) color features) and texture features (GLCM and Law’s texture features) of the ZSS, ZMS, and HAS samples were analyzed via a CV system. Moreover, the fire–ice ion dimensionality reduction algorithm is used to analyze these data by effectively integrating the color and texture features to achieve the nondestructive and fast differentiation of the ZSS, ZMS, and HAS samples, which provides a scientific basis for the nondestructive and fast classification and quality control of samples in related fields.

## 2. Materials and Methods

### 2.1. Sample Collection

**Figure 1:**
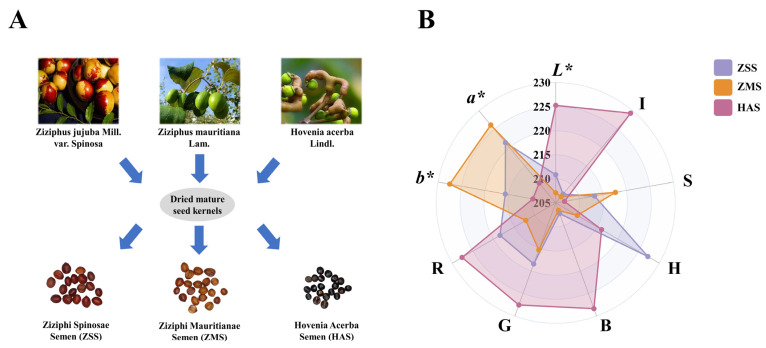
Sample information (**A**) and radar chart of colorimetric values (**B**) of ZSS, ZMS, and HAS. ZSS, Ziziphi Spinosae Semen; ZMS, Ziziphi Mauritianae Semen; HAS, Hovenia Acerba Semen.

A total of 300 batches of ZSS were collected from Hebei, Shaanxi, Shanxi, and Shandong Provinces, with 75 batches from each province. In addition, 88 batches of ZMS and 100 batches of HAS were collected from the herbal market. All the samples were stored in a dry, sealed, and lightproof environment before analysis. Detailed information on the samples is shown in Figure 1A and Table S1. Each sample was given a unique geo-label, such as ZSS-1, ZMS-1 or HAS-1. The latitude and longitude of the samples ranged from 21.08° N to 42.40° N and from 97.31° E to 119.50° E, respectively. The samples were analyzed in the same way. To ensure the authenticity of the samples, all samples were purchased from qualified suppliers or production units. At the same time, the samples were preliminarily analyzed using traditional identification methods (microscopic identification, physical and chemical analysis, etc.), and experts in the field of food identification were invited to confirm the authenticity of the samples. Through the above steps, it was ensured that the samples collected were not adulterated.

### 2.2. Computer Vision-Based Data Collection

#### 2.2.1. Sample Color Value Extraction via the Python Algorithm

In this study, RGB, CIELAB, and HSI were used to analyze the color in samples from ZSS, ZMS, and HAS. The RGB color space represents the color of an image through combinations of the three basic colors, red, green, and blue, and the color intensity value of each pixel can be extracted from it to provide the basic parameters for the color characterization of the image [21]. The CIELAB color space is a color representation that is closer to human eye perception and divides color information into two parts: luminance (L*) and chroma (a* and b*). This color model helps to improve the stability and accuracy of color features under different lighting conditions, making the extracted chromaticity value features more consistent with visual perception. Under this model, different statistical features, such as the mean, variance, and standard deviation, can be used to characterize the color distribution of the samples [22]. The HSI color space, on the other hand, interprets color from the perspective of human vision and is able to efficiently separate the hue, saturation, and luminance information of color [23]. By extracting the key parameters in the HSI model, the color composition of an image and its effect on visual perception can be better understood.

The 4392 datasets (1 detection * 488 batches * 9 categories) used in this study provide a rich sample resource for chroma feature extraction and comparison. Please refer to Table S2 for the specific chromaticity value feature extraction steps.

#### 2.2.2. Sample Texture Extraction via the Python Algorithm

In this study, a gray level coevolution matrix (GLCM) with Law’s texture feature extraction technique was used to analyze the texture information in the ZSS, ZMS, and HAS samples. The GLCM is a widely used technique in image processing that creates a matrix for describing the texture features of an image by counting the frequency of gray-level occurrences of pairs of neighboring pixels in the image [24]. To compute the GLCM, specific orientations (horizontal, vertical, and diagonal) and distances need to be preselected, and the image is analyzed pixel by pixel to record the gray-level frequencies of neighboring pixels around each pixel. On the basis of the generated gray-level covariance matrix, various statistical features, such as energy, contrast, correlation, and entropy, can be extracted, which helps to reveal the intrinsic texture structure of the image. In addition, Law’s texture feature effectively captures the local texture patterns in the image through a series of convolutional kernel operations [25]. After applying convolution kernels with different directions and scales, various types of texture features can be extracted, and these features are usually highly effective in image classification and analysis.

The 10,736 datasets (1 detection * 488 batches * 22 categories) used in this study provide rich samples to support texture feature extraction and comparison. The specific texture extraction steps are detailed in Table S3.

### 2.3. Fire–Ice Ion Dimension Reduction Algorithm

#### 2.3.1. Fire-Ion Dimensionality Reduction Algorithm

In this study, a fire ion dimensionality reduction algorithm based on chromaticity value parameters is proposed to achieve the effective extraction of sample color features and subsequent data fusion dimensionality reduction to support further data processing and research. The code structure consists of several modules, and the necessary libraries, including os, cv2, numpy, and pandas, are first imported to lay the foundation for image analysis. In particular, the study converts the input images into RGB, CIELAB, and HSI color spaces and calculates the mean and standard deviation for each channel. These extracted color features become the key basis for the subsequent data fusion dimensionality reduction process. Furthermore, the fire ion dimensionality reduction function is implemented by the extract_fire_foreground function, which comprehensively utilizes the information of the RGB, CIELAB, and HSI color spaces and adopts the Otsu algorithm to binarize the image to identify the fire feature region effectively. Finally, the fire_detection function integrates foreground extraction and color feature extraction to generate fire feature images and completes the tasks of sample color feature extraction and data fusion dimensionality reduction. In summary, in this study, a systematic approach to fire detection and chromaticity value feature extraction is provided, and chromaticity value parameters are emphasized in the process of image analysis and dimensionality reduction. Table S2 shows the specific steps of the fire ion dimensionality reduction algorithm.

#### 2.3.2. Ice-Ion Dimensionality Reduction Algorithm

In this study, an ice ion downscaling algorithm based on texture feature parameters is constructed with the aim of effectively detecting frozen regions. The algorithm identifies the ice regions by extracting the texture features of the image, including the gray level covariance matrix (GLCM) and Law’s features, and saves the results in an Excel file. The code starts by importing several necessary Python libraries, such as cv2, numpy, pandas, os, matplotlib.pyplot, and skimage.feature, and contains several functional modules: calculate_glcm is used to compute the covariance matrix features of the grayscale image to reflect the texture information; apply_ laws_kernels defines Law’s texture energy kernels via convolution to extract features; and extract_ice_foreground combines the GLCM with Law’s features to generate frozen feature representations. Finally, the ice_detection function integrates foreground extraction and texture feature extraction to generate the ice feature image, which accomplishes the tasks of sample texture feature extraction and data fusion for dimensionality reduction. In summary, a systematic, algorithmic framework is provided for the detection of ice and extraction of texture features, as well as a discussion of how chromaticity values play a significant role in reducing dimensionality through image analysis. Table S3 shows the specific steps of the ice ion dimensionality reduction algorithm.

### 2.4. Discriminative Models

In this paper, 4 machine learning algorithms, including BP (Back Propagation Neural Network), SVM (Support Vector Machine), DBN (Deep Belief Network), and RF (Random Forest), are developed to extract the most effective information and improve classification accuracy. In short, BP is a multi-layer feed-forward network trained using Back Propagation to minimize output errors by adjusting weights and biases [26]. SVM is a supervised model used for classification and regression, which finds the optimal hyperplane to maximize spacing between classes [20]. DBN is a deep learning model made of stacked Restricted Boltzmann Machines, capable of extracting high-level features for complex pattern recognition [27]. RF is an ensemble method that uses multiple decision trees for classification or regression, offering high accuracy and resistance to overfitting, which is suitable for large datasets and high-dimensional features [28]. Meanwhile, when using the above four machine learning algorithms, the samples were divided into a 70% training set and a 30% test set to construct the classification model. The training set was used to train the model and adjust the model parameters through the optimization algorithm to minimize the error, while the test set was used to evaluate the generalization ability and classification performance of the model.

### 2.5. Data Analysis

For the extraction of color and texture features, Python software 3.6.8 (Waverley Software, Palo Alto, CA, USA) was used. The mean ± standard deviation (SD) was used to express the data. An analysis of the data was performed using SPSS software (version 22, SPSS Inc., Chicago, IL, USA). Analysis of variance was used to analyze the data, and Duncan’s multiple-range test and the least significant difference test were used to identify statistically significant differences (*p* < 0.05). Simca software (version 14.1, Sartorius Group, SW, Göttingen, Germany) was used to perform principal component analysis, partial least squares discrimination analysis, and variable importance for projection analysis. Plots were generated via GraphPad Prism (version 9, GraphPad Software, San Diego, CA, USA) and Origin software (version 2021, Origin Lab Corporation, Northampton, MA, USA).

## 3. Results and Discussion

### 3.1. Color Analysis of ZSS, ZMS and HAS

Chromaticity space is a mathematical model for describing colors and is widely used in color science, image processing, and related fields. The RGB, CIELAB, and HSI chromaticity spaces are the three most commonly used representations [21]. On the basis of the above principles and the use of the chromaticity space, radar plots of the RGB, CIELAB, and HSI chromaticity values of the ZSS, ZMS, and HAS samples were constructed, and the results are shown in Figure 1B. As shown in the figure, for the determination of the L* value, the HAS sample has the highest brightness, followed by ZSS and, finally, ZMS. These results indicate that the HAS sample has high brightness under light conditions, which may be related to its surface properties and light reflection ability. Second, for the a* measurements, the red color values of the ZMS samples were significantly greater than those of the other samples, especially for ZSS and HAS. This phenomenon reflects the stronger performance of the ZMS samples in the red spectrum. Similarly, the b* values also support the dominance of ZMS, indicating that the sample is more prominent in the yellow spectrum. After further analysis of the RGB color space, the values of the red (R), green (G), and blue (B) channels all indicated that the HAS sample outperformed the ZSS in terms of the combination of color saturation and luminance, whereas the ZSS in turn outperformed the ZMS. This result suggests that the HAS sample’s superior performance in terms of visual luminance and color saturation gives it an advantage in application scenarios that require a high level of color expression. Moreover, in the HSI chromaticity space, the hue (H) measurements show that the ZSS outperforms the HAS and ZMS, which may be related to the tonal characteristics and hue distribution of the ZSS samples, implying the consistency and sharpness of their hues in visual presentation. In the saturation (S) measurement, ZMS performed the best, showing that this sample has a clear advantage in terms of color vibrancy. Finally, the HAS samples also presented higher values for brightness (I), further confirming their superiority in terms of brightness and color performance [23]. The colorimetric parameters revealed significant differences in color characteristics between the samples.

The combined application of the RGB, CIELAB, and HSI colorimetric systems enabled a comprehensive and systematic analysis of the color properties of the samples. The results provide a scientific basis for an in-depth understanding of the visual performance of the samples.

### 3.2. Texture Characterization of ZSS, ZMS, and HAS

#### 3.2.1. GLCM Texture Characterization

**Figure 2:**
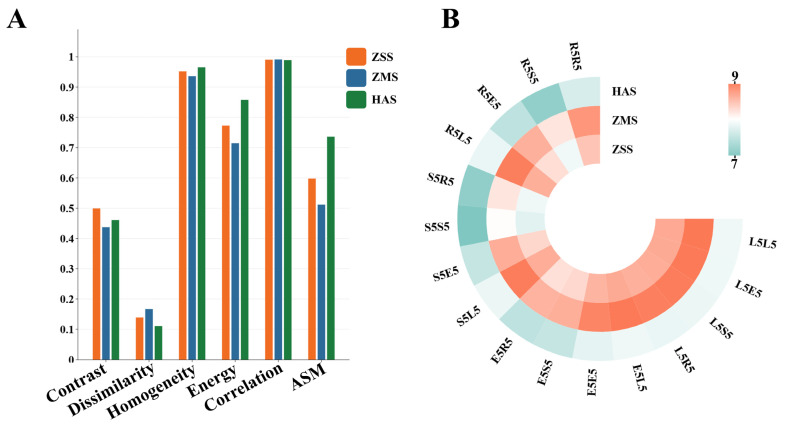
GLCM texture parameter histogram (**A**) and Law’s texture parameter heatmap (**B**) of ZSS, ZMS, and HAS. ZSS, Ziziphi Spinosae Semen; ZMS, Ziziphi Mauritianae Semen; HAS, Hovenia Acerba Semen.

The GLCM is a method that can characterize the texture of an image by calculating the statistical information between different gray levels. Among them, contrast is the contrast, dissimilarity is the difference, homogeneity is the similarity, energy is the energy, correlation is the correlation between pixels, and ASM is the measure of texture thickness. These parameters can help analyze and understand the texture features of an image, thus deepening our understanding and application of image processing and analysis [29,30]. A histogram of the GLCM feature parameters extracted from the ZSS, ZMS, and HAS samples is shown in Figure 2A.

The contrast results show that the ZSS sample has the highest contrast value in the order of ZSS > HAS > ZMS. Contrast is used as a measure of the degree of variation in the gray level distribution in an image, and higher values indicate more distinct textures and more prominent details in the sample. The ZSS sample may exhibit a greater texture contrast under light conditions because of its unique surface features. The dissimilarity results show that the ZMS sample has the highest dissimilarity value, with ZMS > ZSS > HAS. Dissimilarity measures the degree of variation between gray levels in an image, with smaller values indicating a more homogeneous texture profile, and the higher dissimilarity of the ZMS sample may imply that the texture profile of the surface is relatively complex, with a large amount of variation in the gray levels. The homogeneity results show that the HAS sample has the highest homogeneity value, with the result of HAS > ZSS > ZMS. Homogeneity refers to the smoothness of the image texture; the larger the value is, the more uniform the grayscale distribution of the image and the smoother the texture is, and the higher homogeneity of the HAS sample may be related to the smoothness of its surface, which makes it appear more consistent in terms of visual effect. The results show that the energy value of the HAS sample is also significantly greater than that of the other samples, with HAS > ZSS > ZMS. Energy is obtained by calculating the sum of the squares of the elements of the GLCM, which is a parameter reflecting the complexity of the texture of the image. Samples with higher energy values usually have richer textures, and the superiority of the energy value of the HAS sample suggests that it is relatively more vibrant in terms of its texture [31]. The correlation results show that the HAS samples have the highest correlation values with HAS > ZMS > ZSS. Correlation measures the directionality and consistency of the texture in an image, with higher values indicating the greater directionality of the texture structures in the samples, and the high correlation of the HAS samples suggests that the samples are able to better represent consistent texture directional information. The ASM results show that the HAS samples also have higher angular second-order moments (HAS > ZSS > ZMS), which is an important parameter of the GLCM for measuring the texture uniformity of an image [32], and a higher value means that the texture and structure of the samples are more consistent and stable. The high ASM values of the HAS samples further confirm the consistency of the texture features of the HAS samples.

#### 3.2.2. Law’s Texture Characterization

Law’s texture characterization is a convolutional filter-based approach designed to extract texture information from the images [33,34]. The texture analysis model proposed by Law uses a series of filter combinations (luminance, edges, texture, and reflections), which can effectively identify and characterize the subtle structural features in an image by analyzing the image in different directions and scales. The following are the specific meanings of each parameter: L (luminance): luminance is mainly used to capture the basic luminance features of an image and is suitable for characterizing smooth and uniform textures. E (edge): this is used to emphasize the boundaries and high contrast areas in an image, which is effective in capturing texture variations. S (texture): texture focuses on subtle surface variations, emphasizing the roughness of the surface texture. R (reflection): reflection characterizes the light reflection of an image and properties suitable for characterizing high gloss or reflective materials.

A heatmap of texture parameters was constructed on the basis of the results of analyzing the three samples, ZSS, ZMS, and HAS, in terms of their texture parameters (16 parameters in total), and the results are shown in Figure 2B. The figure shows that the specific results of the ZSS, ZMS, and HAS samples in terms of the values of L5L5, L5E5, L5S5, L5R5, E5L5, E5E5, E5S5, E5R5, S5L5, S5E5, S5S5, S5R5, R5L5, R5E5, R5S5, and R5R5 are as follows: ZMS > ZSS > HAS. The specific analyses are given as follows: brightness characteristics (L series): ZMS has the highest L5L5, L5E5, and L5S5 values, indicating that its surface brightness is outstanding, which can effectively reflect light, enhance visual attractiveness, and show rich details under different lighting conditions. Edge characterization (E-series): the E5E5 value of the ZMS shows a clear advantage in edge detection, helping to clearly depict the sample shape and details. Texture characteristics (S-series): the performance of ZMS in terms of the S5S5 parameter highlights the complexity and uniformity of its surface texture. Reflection characteristics (R-series): ZMS shows higher reflectance in R5R5, R5E5, and R5S5, indicating a stronger sense of gloss.

### 3.3. Analysis of the Fire–Ice Ion Downscaling Results from ZSS, ZMS, and HAS

**Figure 3:**
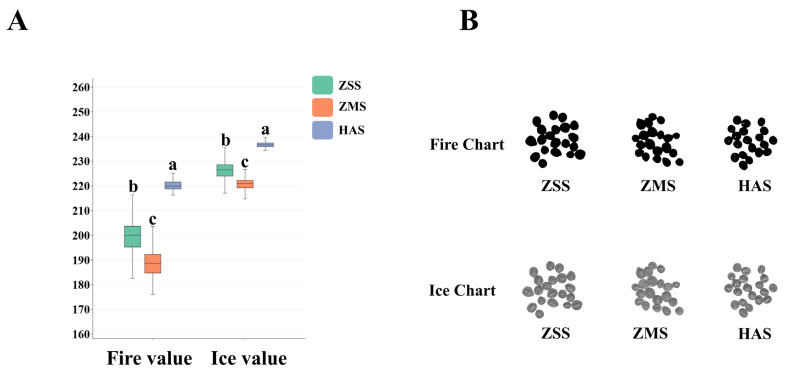
Fire–ice value box chart (**A**) and fire–ice chart (**B**) of ZSS, ZMS, and HAS. The letters (a–c) above the bars indicate significant differences as determined by Duncan’s multiple-range test (*p* < 0.05). ZSS, Ziziphi Spinosae Semen; ZMS, Ziziphi Mauritianae Semen; HAS, Hovenia Acerba Semen.

The research team independently developed an algorithm to reduce fire–ice ion dimensionality that integrates color and texture features to enhance the differentiation of ZSS, ZMS, and HAS samples. By utilizing RGB, CIELAB, and HSI color space parameters, the algorithm constructs a “fire value” index, which can effectively simplify and extract color characteristics. The algorithm also analyzes the texture features and structural data of the sample surface at depth, including the GLCM and Law’s texture features, to extract the “ice value” index. The fire value results obtained for the three seed samples, ZSS, ZMS, and HAS, after applying the fire ion downscaling algorithm as well as the ice ion downscaling algorithm, all indicate that HAS > ZSS > ZMS (Figure 3A).

The fire ion downscaling algorithm effectively simplifies and integrates the color characteristics of each sample by integrating RGB, CIELAB, and HSI chromaticity space parameters [35]. Specifically, in the CIELAB color space, the main advantage of the CIELAB space is its color model, which is based on human visual perception. The model divides colors into luminance (L*) and chroma (a* and b*), allowing the color characteristics of the samples to be more intuitively reflected in the perception of the human eye. Higher values of this parameter indicate that the sample is more visually appealing and expressive. RGB color space: the RGB color space defines color through the mixing of red, green, and blue primary colors and is suitable for use in digital image processing. Through the fusion and analysis of the RGB space, the fire value obtained can better capture the basic color composition of the sample and reflect the color performance ability of the sample under different lighting conditions. HSI color space: the HSI color space focuses on the combination of hue, saturation, and luminance, which is closer to the human eye’s true feeling of color. Therefore, a comprehensive analysis of HSI parameters can provide a more in-depth and comprehensive view of the fire performance of a sample. Combining the analysis of these three chromaticity spatial parameters, the results show that the HAS sample has the highest overall score in terms of fire value, reflecting its significant advantages in terms of color saturation, brightness, visual attractiveness, etc. The ZSS sample performs moderately well, and although its fire value is higher than that of the ZMS sample, it still needs to be improved in terms of its color performance and visual effect, while the ZMS sample scores the lowest score, which indicates that there is room for improvement in terms of color hierarchy and visual consistency. This consistency indicates that there is room for improvement.

Compared with the fire ion algorithm, the ice ion downscaling algorithm analyzes the texture characteristics and structural information of samples at depth by combining the gray level covariance matrix (GLCM) and Law’s texture features [36]. GLCM: this method calculates the spatial relationship of pixel gray levels in the image and helps to analyze the texture characteristics of the samples by extracting features such as contrast, uniformity, and entropy. This algorithm is particularly suitable for describing the complexity of the texture and consistency of a sample’s surface, providing a scientific basis for subsequent visual analysis. Law’s texture characterization: this is a technique for capturing texture information through a filter, where Law’s characterization emphasizes variations in both overall and local patterns. This allows for a more comprehensive description of the fine structure of the sample surface, which, in turn, enhances the understanding of the texture features. The results of the ice ion downscaling algorithm show that the HAS sample scores the highest in texture features, which indicates a finer and more uniform surface structure that provides a better appearance and tactile experience. The ZSS sample performs relatively consistently, indicating better texture quality and an acceptable visual effect. The ZMS sample continues to exhibit underperformance, reflecting its texture uniformity and deficiencies in capturing detail.

Figure 3B shows the fire–ice foreground images generated from the processing of the ZSS, ZMS, and HAS samples via the fire–ice ion downscaling algorithm. The fire–ice foreground image is a binary image that shows the distributions of the ZSS, ZMS, and HAS samples and reveals clear regional consistency, where the samples are distinctly different from the surrounding backgrounds, which can be recognized with a high degree of accuracy. The effectiveness of the detection algorithm can be verified by visualizing these images.

### 3.4. Multivariate Statistical Analysis

#### 3.4.1. Raw Color and Texture Characterization of ZSS, ZMS, and HAS

**Figure 4:**
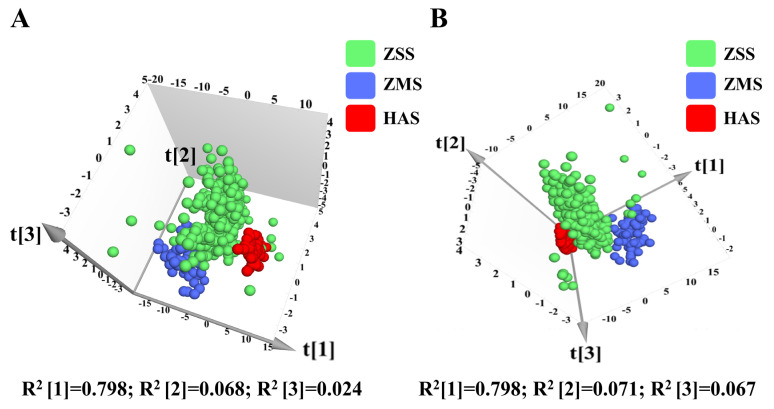
Score plots of the PCA model for ZSS, ZMS, and HAS of raw color and texture characterization (**A**); score plots of the PLS-DA model for ZSS, ZMS, and HAS of raw color and texture characterization (**B**). ZSS, Ziziphi Spinosae Semen; ZMS, Ziziphi Mauritianae Semen; HAS, Hovenia Acerba Semen. PCA, principal component analysis; PLS-DA, partial least squares discrimination analysis.

To achieve the nondestructive and fast differentiation of the three samples (ZSS, ZMS, and HAS), a multivariate statistical approach was used to analyze the chromaticity and texture features of the samples in this study. Principal component analysis (PCA), a commonly used unsupervised dimensionality reduction method, has been widely used in recent years to distinguish varieties and geographical origins of agricultural products [37,38]. The chromaticity values of the ZSS, ZMS, and HAS samples were analyzed with texture feature parameters via principal component analysis (PCA) to reveal intra- and intergroup variability. The results of the data analysis revealed that the first, second, and third principal components accounted for 79.8%, 6.8%, and 2.4% of the variance, respectively, with a cumulative contribution of 89.0%, as shown in Figure 4A. The ZSS, ZMS, and HAS samples presented different clustering patterns in the PCA score plots, and overall, the samples could be clearly categorized into three regions. However, the separation between the ZSS sample groups was poor, and some of the samples overlapped with the clustered regions of the ZMS and HAS samples; moreover, the degree of clustering within the ZSS sample groups was low, and the whole showed a large degree of dispersion.

**Figure 5:**
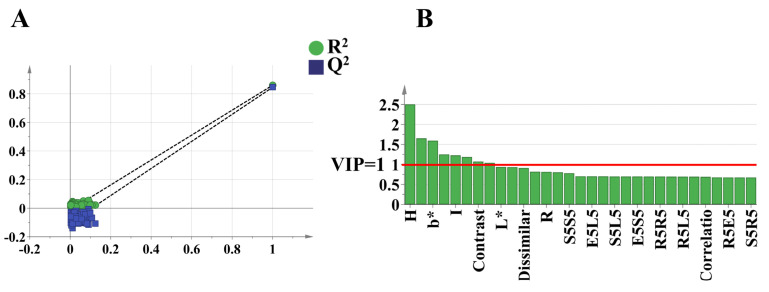
Cross-validation results with 200 calculations using a permutation test for ZSS, ZMS, and HAS of raw color and texture characterization (**A**); VIP plots for ZSS, ZMS, and HAS of raw color and texture characterization (**B**). ZSS, Ziziphi Spinosae Semen; ZMS, Ziziphi Mauritianae Semen; HAS, Hovenia Acerba Semen. VIP, variable importance for projecting.

Unlike PCA, partial least squares discriminant analysis (PLS-DA) is an effective supervised statistical analysis method [39]. PLS-DA can effectively filter out overlapping information and identify feature indicators with significant grouping contributions by evaluating the importance of variables in the principal components [40]. Figure 4B shows the results of the PLS-DA analysis, in which the discrimination rates of t [1], t [2], and t [3] were 79.8%, 7.1%, and 6.7%, respectively, and the cumulative contribution rate reached 93.6%. Compared with the clustering results of PCA, PLS-DA significantly improved the 3D fractional scatterplot clustering of the ZSS, ZMS, and HAS samples, with reduced dispersion within the sample clusters and increased separation between groups. These findings indicate that the PLS-DA model is significantly superior in terms of interference reduction and analytical dimensionality reduction. To prevent overfitting in the modeling process, a permutation test was used to assess the validity of the PLS-DA model. Figure 5A illustrates the results of the replacement test, where the reduced replacement retention is negatively correlated with the R² and Q² values of the stochastic model, indicating that the initial model is not overfitted and demonstrates its robustness. In addition, the variable importance in projection (VIP) values [41] obtained from the PLS-DA model was used to measure the strength of the influence of the chromaticity values and texture features of the ZSS, ZMS, and HAS samples on the classification of each group of samples. It is usually considered that feature factors with VIP values greater than one contribute significantly to the model. In this study, the characterization factors with VIP > 1 and *p* < 0.05 were identified as significantly different factors, which led to the screening of eight key trait factors, which included H, a*, b*, B, I, S, contrast, and homogeneity (Figure 5B). These characterization factors provide an important reference for the further exploration of sample classification and its biological significance.

#### 3.4.2. Characterization Data Analysis After Dimensionality Reduction Processing of Fire–Ice Ions for ZSS, ZMS, and HAS

**Figure 6:**
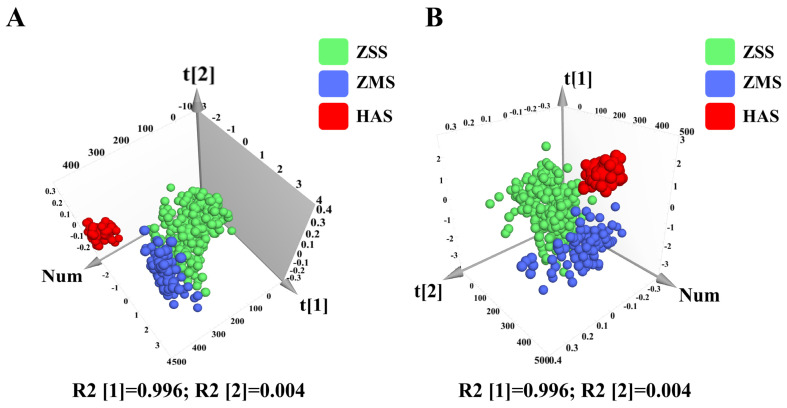
Score plots of the PCA model for ZSS, ZMS, and HAS of fire–ice ions dimensionality reduction data (**A**); score plots of the PLS-DA model for ZSS, ZMS, and HAS of fire–ice ions dimensionality reduction data (**B**). ZSS, Ziziphi Spinosae Semen; ZMS, Ziziphi Mauritianae Semen; HAS, Hovenia Acerba Semen. PCA, principal component analysis; PLS-DA, partial least squares discrimination analysis.

Moreover, a new database was created for multivariate statistical analysis on the basis of the relationship between the fire values (including chromaticity values) and ice values (including texture features) of the ZSS, ZMS, and HAS samples collected via the fire–ice ion downscaling algorithm. The fusion features of fire values and ice values provide an important basis for sample differentiation. Principal component analysis (PCA) was performed on the fire and ice values of the ZSS, ZMS, and HAS samples, and the results of the analysis are shown in Figure 6A. The PCA results revealed that the first and second principal components accounted for 99.6% and 0.4% of the variance, respectively, with a cumulative contribution of 100.0%. Compared with the dataset before dimensionality reduction, the method significantly improved the overall discrimination between the samples. The different sample groups showed clear clustering patterns in the PCA score plots, with a high degree of separation between the samples and no overlap in the aggregation regions of the different sample groups. Moreover, the degree of aggregation within each sample group was high, and no discrete samples were observed.

**Figure 7:**
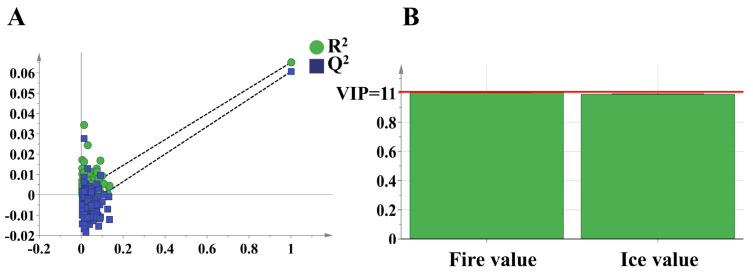
Cross-validation results with 200 calculations using a permutation test for ZSS, ZMS, and HAS of fire–ice ions dimensionality reduction data (**A**); VIP plots for ZSS, ZMS, and HAS of fire–ice ions dimensionality reduction data (**B**). ZSS, Ziziphi Spinosae Semen; ZMS, Ziziphi Mauritianae Semen; HAS, Hovenia Acerba Semen. VIP, variable importance for projecting.

The results of the PLS-DA analysis are shown in Figure 6B, in which the discrimination rates of t [1] and t [2] are 99.6% and 0.4%, respectively, and the cumulative contribution rate is also 100.0%. Similarly to the PCA results, the three-dimensional fractional scatterplot clustering analysis of the ZSS, ZMS, and HAS samples by PLS-DA revealed that the clustering effect of the samples was significantly improved after the dimensionality reduction process, with less dispersion within the sample clusters and more separation between the groups. This suggests that PLS-DA successfully reduces interference and lowers the dimensionality of the analysis, which, in turn, enhances the validity of the model. The PLS-DA model demonstrates good adaptability and predictive power in tracing the origins of samples. To assess the validity of the supervised model and prevent overfitting, a permutation test was used. Figure 7A shows the replacement test plot of the PLS-DA model, which is negatively correlated with the R² and Q² values of the stochastic model, indicating that the initial model is not overfitted and shows its robustness. In addition, the VIP values generated by the PLS-DA model were used to measure the strength of the effect of fire values versus ice values on the classification of each group of samples and to explore biologically significant differential traits. As shown in Figure 7B, the characterization factors with VIP values greater than one and *p* < 0.05 were identified as significantly different factors in this study, and the fire value was finally selected as a potential characterization trait factor to differentiate among the ZSS, ZMS, and HAS samples.

### 3.5. Reverse Validation Analysis Based on Machine Learning Classification Algorithms

**Figure 8:**
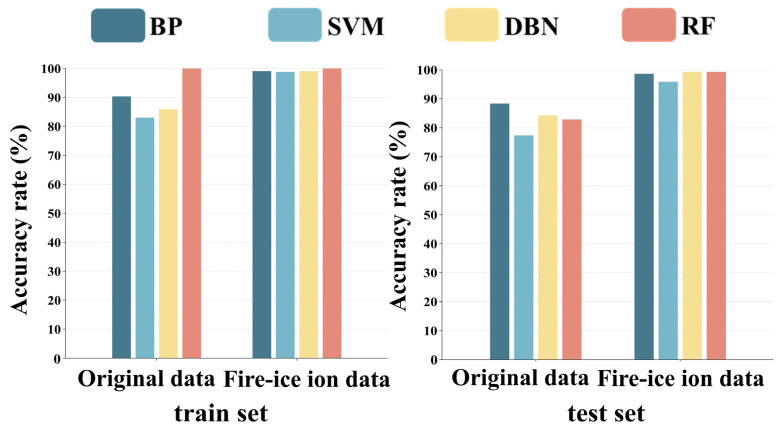
Evaluation metrics of 4 machine learning algorithms (BP, SVM, DBN, and RF).

In order to verify the accuracy and reliability of the results, four machine learning algorithms, BP, SVM, DBN, and RF, were developed for further analysis. Data matrices were constructed using original data (chromaticity values and texture parameters) and fire–ice ion data, respectively, which were then imported into the MATLAB 2020b software (MathWorks, Natick, MA, USA) to create these models. Accuracy is an important metric for assessing the performance of the algorithmic models. The results in Figure 8 show that in the data matrices constructed with the original data (chromaticity values and texture parameters) from ZSS, ZMS, and HAS, the training accuracies of the BP, SVM, DBN, and RF classifiers were 90.35%, 83.04%, 85.96%, and 100.00%, respectively. And the testing accuracies are 88.36%, 77.40%, 84.25%, and 82.88%. In the data matrix constructed with fire–ice ion data from ZSS, ZMS, and HAS, the training accuracies of BP, SVM, DBN, and RF classifiers were 99.12%, 98.83%, 99.12%, and 100.00%. And the testing accuracies were 98.63%, 95.89%, 99.32%, and 99.32%, respectively. This result shows that the reduced dimensionality data can be better classified, and the learning effect of the model can be significantly improved. Meanwhile, the graphs based on the confusion matrix are shown in Figure S1. Obviously, in the data matrix constructed with the original data (chromaticity values and texture parameters) from ZSS, ZMS, and HAS, the BP model shows better discrimination accuracy, while in the data matrix constructed with the fire–ice ion data from ZSS, ZMS, and HAS, the RF model shows better discrimination accuracy. In addition, the convergence speed of the model is significantly improved due to the reduction in data volume after dimensionality reduction. In summary, using the fire–ice dimensionality reduction algorithm significantly enhances the classification accuracy and convergence efficiency of machine learning algorithms. This finding is particularly valuable for practical applications involving high-dimensional data.

## 4. Conclusions

The color and texture features of the ZSS, ZMS, and HAS samples were analyzed via RGB, CIELAB, and HSI color spaces and GLCM and Law’s texture features. Significant differences were found among the three samples. To improve the differentiation ability of the samples, the fire–ice ion dimensionality reduction algorithm was applied. As a result of PCA and PLS-DA, the algorithm was able to differentiate HAS samples from ZSS and ZMS samples. However, the VIP analysis revealed the fire value as the main characterization factor (VIP > 1, *p* < 0.05), which could be used to effectively differentiate the three samples. This establishes a scientific foundation for nondestructive rapid classification and quality control. The algorithm-processed data, when input into a BP neural network classification model, achieved a 100% discrimination rate and improved the convergence speed.

On the basis of computer vision and fire–ice ion dimensionality reduction algorithms, ZSS, ZMS, and HAS samples were identified nondestructively and rapidly as a result of this study, providing a scientific basis for sample classification and quality control. This method has the advantage of being rapid, nondestructive, and inexpensive, which provides an effective supplement to the limitations of traditional detection methods.

## Data Availability

The original contributions presented in the study are included in the article/Supplementary Material, further inquiries can be directed to the corresponding authors.

## Supplementary Materials

The following supporting information can be downloaded at: https://www.mdpi.com/article/10.3390/foods14010005/s1, Table S1: Geographic region information for ZSS, ZMS and HAS; Table S2: Fire ion dimensionality reduction algorithm based on chromaticity value feature extraction; Table S3: Ice ion dimensionality reduction algorithm based on texture feature extraction; Figure S1: Confusion matrices of the train set (A) and test set (B) of 4 machine learning algorithms based on the original data for classification discrimination; Confusion matrices of the training set (C) and test set (D) of 4 machine learning algorithms based on the fire-ice ion data for classification discrimination.

## Abbreviations

ZSS, Ziziphi Spinosae Semen; ZMS, Ziziphi Mauritianae Semen; HAS, Hovenia Acerba Semen; GLCM, gray level co-occurrence matrix; PCA, principal component analysis; PLS-DA, partial least squares discriminant analysis; VIP-analysis, variable importance in projection analysis; MDF, multivariate data fusion.
