# Supplementary material for: Computer Vision-Based Fire–Ice Ion Algorithm for Rapid and Nondestructive Authentication of Ziziphi Spinosae Semen and Its Counterfeits
Source: Foods. 2024 Dec 24;14(1):5. doi: 10.3390/foods14010005 (PMC11719666; doi:10.3390/foods14010005)
Supplement: Supplementary file 1 [file foods-14-00005-s001.zip › foods-3354418-supplementary.docx]

**Table. S1** Geographic region information for ZSS, ZMS and HAS.

| NO. | Name | Abbreviations | Province | Longitude  (˚E) | Latitude  (˚N) | Altitude  (m) | Batch number |
| --- | --- | --- | --- | --- | --- | --- | --- |
| 1 | ZSS | HB | Hebei | 113.27~ 119.50 | 36.05~42.40 | 20~2882 | 220203101 |
| 2 | ZSS | HB | Hebei | 113.27~ 119.50 | 36.05~42.40 | 20~2882 | 220203102 |
| 3 | ZSS | HB | Hebei | 113.27~ 119.50 | 36.05~42.40 | 20~2882 | 220203103 |
| 4 | ZSS | HB | Hebei | 113.27~ 119.50 | 36.05~42.40 | 20~2882 | 220203104 |
| 5 | ZSS | HB | Hebei | 113.27~ 119.50 | 36.05~42.40 | 20~2882 | 220203105 |
| 6 | ZSS | HB | Hebei | 113.27~ 119.50 | 36.05~42.40 | 20~2882 | 220203106 |
| 7 | ZSS | HB | Hebei | 113.27~ 119.50 | 36.05~42.40 | 20~2882 | 220203107 |
| 8 | ZSS | HB | Hebei | 113.27~ 119.50 | 36.05~42.40 | 20~2882 | 220203108 |
| 9 | ZSS | HB | Hebei | 113.27~ 119.50 | 36.05~42.40 | 20~2882 | 220203109 |
| 10 | ZSS | HB | Hebei | 113.27~ 119.50 | 36.05~42.40 | 20~2882 | 220203110 |
| 11 | ZSS | HB | Hebei | 113.27~ 119.50 | 36.05~42.40 | 20~2882 | 220203111 |
| 12 | ZSS | HB | Hebei | 113.27~ 119.50 | 36.05~42.40 | 20~2882 | 220203112 |
| 13 | ZSS | HB | Hebei | 113.27~ 119.50 | 36.05~42.40 | 20~2882 | 220203113 |
| 14 | ZSS | HB | Hebei | 113.27~ 119.50 | 36.05~42.40 | 20~2882 | 220203114 |
| 15 | ZSS | HB | Hebei | 113.27~ 119.50 | 36.05~42.40 | 20~2882 | 220203115 |
| 16 | ZSS | HB | Hebei | 113.27~ 119.50 | 36.05~42.40 | 20~2882 | 220203201 |
| 17 | ZSS | HB | Hebei | 113.27~ 119.50 | 36.05~42.40 | 20~2882 | 220203202 |
| 18 | ZSS | HB | Hebei | 113.27~ 119.50 | 36.05~42.40 | 20~2882 | 220203203 |
| 19 | ZSS | HB | Hebei | 113.27~ 119.50 | 36.05~42.40 | 20~2882 | 220203204 |
| 20 | ZSS | HB | Hebei | 113.27~ 119.50 | 36.05~42.40 | 20~2882 | 220203205 |
| 21 | ZSS | HB | Hebei | 113.27~ 119.50 | 36.05~42.40 | 20~2882 | 220203206 |
| 22 | ZSS | HB | Hebei | 113.27~ 119.50 | 36.05~42.40 | 20~2882 | 220203207 |
| 23 | ZSS | HB | Hebei | 113.27~ 119.50 | 36.05~42.40 | 20~2882 | 220203208 |
| 24 | ZSS | HB | Hebei | 113.27~ 119.50 | 36.05~42.40 | 20~2882 | 220203209 |
| 25 | ZSS | HB | Hebei | 113.27~ 119.50 | 36.05~42.40 | 20~2882 | 220203210 |
| 26 | ZSS | HB | Hebei | 113.27~ 119.50 | 36.05~42.40 | 20~2882 | 220203211 |
| 27 | ZSS | HB | Hebei | 113.27~ 119.50 | 36.05~42.40 | 20~2882 | 220203212 |
| 28 | ZSS | HB | Hebei | 113.27~ 119.50 | 36.05~42.40 | 20~2882 | 220203213 |
| 29 | ZSS | HB | Hebei | 113.27~ 119.50 | 36.05~42.40 | 20~2882 | 220203214 |
| 30 | ZSS | HB | Hebei | 113.27~ 119.50 | 36.05~42.40 | 20~2882 | 220203215 |
| 31 | ZSS | HB | Hebei | 113.27~ 119.50 | 36.05~42.40 | 20~2882 | 220203301 |
| 32 | ZSS | HB | Hebei | 113.27~ 119.50 | 36.05~42.40 | 20~2882 | 220203302 |
| 33 | ZSS | HB | Hebei | 113.27~ 119.50 | 36.05~42.40 | 20~2882 | 220203303 |
| 34 | ZSS | HB | Hebei | 113.27~ 119.50 | 36.05~42.40 | 20~2882 | 220203304 |
| 35 | ZSS | HB | Hebei | 113.27~ 119.50 | 36.05~42.40 | 20~2882 | 220203305 |
| 36 | ZSS | HB | Hebei | 113.27~ 119.50 | 36.05~42.40 | 20~2882 | 220203306 |
| 37 | ZSS | HB | Hebei | 113.27~ 119.50 | 36.05~42.40 | 20~2882 | 220203307 |
| 38 | ZSS | HB | Hebei | 113.27~ 119.50 | 36.05~42.40 | 20~2882 | 220203308 |
| 39 | ZSS | HB | Hebei | 113.27~ 119.50 | 36.05~42.40 | 20~2882 | 220203309 |
| 40 | ZSS | HB | Hebei | 113.27~ 119.50 | 36.05~42.40 | 20~2882 | 220203310 |
| 41 | ZSS | HB | Hebei | 113.27~ 119.50 | 36.05~42.40 | 20~2882 | 220203311 |
| 42 | ZSS | HB | Hebei | 113.27~ 119.50 | 36.05~42.40 | 20~2882 | 220203312 |
| 43 | ZSS | HB | Hebei | 113.27~ 119.50 | 36.05~42.40 | 20~2882 | 220203313 |
| 44 | ZSS | HB | Hebei | 113.27~ 119.50 | 36.05~42.40 | 20~2882 | 220203314 |
| 45 | ZSS | HB | Hebei | 113.27~ 119.50 | 36.05~42.40 | 20~2882 | 220203315 |
| 46 | ZSS | HB | Hebei | 113.27~ 119.50 | 36.05~42.40 | 20~2882 | 220203401 |
| 47 | ZSS | HB | Hebei | 113.27~ 119.50 | 36.05~42.40 | 20~2882 | 220203402 |
| 48 | ZSS | HB | Hebei | 113.27~ 119.50 | 36.05~42.40 | 20~2882 | 220203403 |
| 49 | ZSS | HB | Hebei | 113.27~ 119.50 | 36.05~42.40 | 20~2882 | 220203404 |
| 50 | ZSS | HB | Hebei | 113.27~ 119.50 | 36.05~42.40 | 20~2882 | 220203405 |
| 51 | ZSS | HB | Hebei | 113.27~ 119.50 | 36.05~42.40 | 20~2882 | 220203406 |
| 52 | ZSS | HB | Hebei | 113.27~ 119.50 | 36.05~42.40 | 20~2882 | 220203407 |
| 53 | ZSS | HB | Hebei | 113.27~ 119.50 | 36.05~42.40 | 20~2882 | 220203408 |
| 54 | ZSS | HB | Hebei | 113.27~ 119.50 | 36.05~42.40 | 20~2882 | 220203409 |
| 55 | ZSS | HB | Hebei | 113.27~ 119.50 | 36.05~42.40 | 20~2882 | 220203410 |
| 56 | ZSS | HB | Hebei | 113.27~ 119.50 | 36.05~42.40 | 20~2882 | 220203411 |
| 57 | ZSS | HB | Hebei | 113.27~ 119.50 | 36.05~42.40 | 20~2882 | 220203412 |
| 58 | ZSS | HB | Hebei | 113.27~ 119.50 | 36.05~42.40 | 20~2882 | 220203413 |
| 59 | ZSS | HB | Hebei | 113.27~ 119.50 | 36.05~42.40 | 20~2882 | 220203414 |
| 60 | ZSS | HB | Hebei | 113.27~ 119.50 | 36.05~42.40 | 20~2882 | 220203415 |
| 61 | ZSS | HB | Hebei | 113.27~ 119.50 | 36.05~42.40 | 20~2882 | 220203501 |
| 62 | ZSS | HB | Hebei | 113.27~ 119.50 | 36.05~42.40 | 20~2882 | 220203502 |
| 63 | ZSS | HB | Hebei | 113.27~ 119.50 | 36.05~42.40 | 20~2882 | 220203503 |
| 64 | ZSS | HB | Hebei | 113.27~ 119.50 | 36.05~42.40 | 20~2882 | 220203504 |
| 65 | ZSS | HB | Hebei | 113.27~ 119.50 | 36.05~42.40 | 20~2882 | 220203505 |
| 66 | ZSS | HB | Hebei | 113.27~ 119.50 | 36.05~42.40 | 20~2882 | 220203506 |
| 67 | ZSS | HB | Hebei | 113.27~ 119.50 | 36.05~42.40 | 20~2882 | 220203507 |
| 68 | ZSS | HB | Hebei | 113.27~ 119.50 | 36.05~42.40 | 20~2882 | 220203508 |
| 69 | ZSS | HB | Hebei | 113.27~ 119.50 | 36.05~42.40 | 20~2882 | 220203509 |
| 70 | ZSS | HB | Hebei | 113.27~ 119.50 | 36.05~42.40 | 20~2882 | 220203510 |
| 71 | ZSS | HB | Hebei | 113.27~ 119.50 | 36.05~42.40 | 20~2882 | 220203511 |
| 72 | ZSS | HB | Hebei | 113.27~ 119.50 | 36.05~42.40 | 20~2882 | 220203512 |
| 73 | ZSS | HB | Hebei | 113.27~ 119.50 | 36.05~42.40 | 20~2882 | 220203513 |
| 74 | ZSS | HB | Hebei | 113.27~ 119.50 | 36.05~42.40 | 20~2882 | 220203514 |
| 75 | ZSS | HB | Hebei | 113.27~ 119.50 | 36.05~42.40 | 20~2882 | 220203515 |
| 76 | ZSS | SD | Shandong | 114.36~118.26 | 34.22~38.23 | 0~ 1545 | 21020101 |
| 77 | ZSS | SD | Shandong | 114.36~118.26 | 34.22~38.23 | 0~ 1545 | 21020102 |
| 78 | ZSS | SD | Shandong | 114.36~118.26 | 34.22~38.23 | 0~ 1545 | 21020103 |
| 79 | ZSS | SD | Shandong | 114.36~118.26 | 34.22~38.23 | 0~ 1545 | 21020104 |
| 80 | ZSS | SD | Shandong | 114.36~118.26 | 34.22~38.23 | 0~ 1545 | 21020105 |
| 81 | ZSS | SD | Shandong | 114.36~118.26 | 34.22~38.23 | 0~ 1545 | 21020106 |
| 82 | ZSS | SD | Shandong | 114.36~118.26 | 34.22~38.23 | 0~ 1545 | 21020107 |
| 83 | ZSS | SD | Shandong | 114.36~118.26 | 34.22~38.23 | 0~ 1545 | 21020108 |
| 84 | ZSS | SD | Shandong | 114.36~118.26 | 34.22~38.23 | 0~ 1545 | 21020109 |
| 85 | ZSS | SD | Shandong | 114.36~118.26 | 34.22~38.23 | 0~ 1545 | 21020110 |
| 86 | ZSS | SD | Shandong | 114.36~118.26 | 34.22~38.23 | 0~ 1545 | 21020111 |
| 87 | ZSS | SD | Shandong | 114.36~118.26 | 34.22~38.23 | 0~ 1545 | 21020112 |
| 88 | ZSS | SD | Shandong | 114.36~118.26 | 34.22~38.23 | 0~ 1545 | 21020113 |
| 89 | ZSS | SD | Shandong | 114.36~118.26 | 34.22~38.23 | 0~ 1545 | 21020114 |
| 90 | ZSS | SD | Shandong | 114.36~118.26 | 34.22~38.23 | 0~ 1545 | 21020115 |
| 91 | ZSS | SD | Shandong | 114.36~118.26 | 34.22~38.23 | 0~ 1545 | 21020201 |
| 92 | ZSS | SD | Shandong | 114.36~118.26 | 34.22~38.23 | 0~ 1545 | 21020202 |
| 93 | ZSS | SD | Shandong | 114.36~118.26 | 34.22~38.23 | 0~ 1545 | 21020203 |
| 94 | ZSS | SD | Shandong | 114.36~118.26 | 34.22~38.23 | 0~ 1545 | 21020204 |
| 95 | ZSS | SD | Shandong | 114.36~118.26 | 34.22~38.23 | 0~ 1545 | 21020205 |
| 96 | ZSS | SD | Shandong | 114.36~118.26 | 34.22~38.23 | 0~ 1545 | 21020206 |
| 97 | ZSS | SD | Shandong | 114.36~118.26 | 34.22~38.23 | 0~ 1545 | 21020207 |
| 98 | ZSS | SD | Shandong | 114.36~118.26 | 34.22~38.23 | 0~ 1545 | 21020208 |
| 99 | ZSS | SD | Shandong | 114.36~118.26 | 34.22~38.23 | 0~ 1545 | 21020209 |
| 100 | ZSS | SD | Shandong | 114.36~118.26 | 34.22~38.23 | 0~ 1545 | 21020210 |
| 101 | ZSS | SD | Shandong | 114.36~118.26 | 34.22~38.23 | 0~ 1545 | 21020211 |
| 102 | ZSS | SD | Shandong | 114.36~118.26 | 34.22~38.23 | 0~ 1545 | 21020212 |
| 103 | ZSS | SD | Shandong | 114.36~118.26 | 34.22~38.23 | 0~ 1545 | 21020213 |
| 104 | ZSS | SD | Shandong | 114.36~118.26 | 34.22~38.23 | 0~ 1545 | 21020214 |
| 105 | ZSS | SD | Shandong | 114.36~118.26 | 34.22~38.23 | 0~ 1545 | 21020215 |
| 106 | ZSS | SD | Shandong | 114.36~118.26 | 34.22~38.23 | 0~ 1545 | 21020301 |
| 107 | ZSS | SD | Shandong | 114.36~118.26 | 34.22~38.23 | 0~ 1545 | 21020302 |
| 108 | ZSS | SD | Shandong | 114.36~118.26 | 34.22~38.23 | 0~ 1545 | 21020303 |
| 109 | ZSS | SD | Shandong | 114.36~118.26 | 34.22~38.23 | 0~ 1545 | 21020304 |
| 110 | ZSS | SD | Shandong | 114.36~118.26 | 34.22~38.23 | 0~ 1545 | 21020305 |
| 111 | ZSS | SD | Shandong | 114.36~118.26 | 34.22~38.23 | 0~ 1545 | 21020306 |
| 112 | ZSS | SD | Shandong | 114.36~118.26 | 34.22~38.23 | 0~ 1545 | 21020307 |
| 113 | ZSS | SD | Shandong | 114.36~118.26 | 34.22~38.23 | 0~ 1545 | 21020308 |
| 114 | ZSS | SD | Shandong | 114.36~118.26 | 34.22~38.23 | 0~ 1545 | 21020309 |
| 115 | ZSS | SD | Shandong | 114.36~118.26 | 34.22~38.23 | 0~ 1545 | 21020310 |
| 116 | ZSS | SD | Shandong | 114.36~118.26 | 34.22~38.23 | 0~ 1545 | 21020311 |
| 117 | ZSS | SD | Shandong | 114.36~118.26 | 34.22~38.23 | 0~ 1545 | 21020312 |
| 118 | ZSS | SD | Shandong | 114.36~118.26 | 34.22~38.23 | 0~ 1545 | 21020313 |
| 119 | ZSS | SD | Shandong | 114.36~118.26 | 34.22~38.23 | 0~ 1545 | 21020314 |
| 120 | ZSS | SD | Shandong | 114.36~118.26 | 34.22~38.23 | 0~ 1545 | 21020315 |
| 121 | ZSS | SD | Shandong | 114.36~118.26 | 34.22~38.23 | 0~ 1545 | 21020401 |
| 122 | ZSS | SD | Shandong | 114.36~118.26 | 34.22~38.23 | 0~ 1545 | 21020402 |
| 123 | ZSS | SD | Shandong | 114.36~118.26 | 34.22~38.23 | 0~ 1545 | 21020403 |
| 124 | ZSS | SD | Shandong | 114.36~118.26 | 34.22~38.23 | 0~ 1545 | 21020404 |
| 125 | ZSS | SD | Shandong | 114.36~118.26 | 34.22~38.23 | 0~ 1545 | 21020405 |
| 126 | ZSS | SD | Shandong | 114.36~118.26 | 34.22~38.23 | 0~ 1545 | 21020406 |
| 127 | ZSS | SD | Shandong | 114.36~118.26 | 34.22~38.23 | 0~ 1545 | 21020407 |
| 128 | ZSS | SD | Shandong | 114.36~118.26 | 34.22~38.23 | 0~ 1545 | 21020408 |
| 129 | ZSS | SD | Shandong | 114.36~118.26 | 34.22~38.23 | 0~ 1545 | 21020409 |
| 130 | ZSS | SD | Shandong | 114.36~118.26 | 34.22~38.23 | 0~ 1545 | 21020410 |
| 131 | ZSS | SD | Shandong | 114.36~118.26 | 34.22~38.23 | 0~ 1545 | 21020411 |
| 132 | ZSS | SD | Shandong | 114.36~118.26 | 34.22~38.23 | 0~ 1545 | 21020412 |
| 133 | ZSS | SD | Shandong | 114.36~118.26 | 34.22~38.23 | 0~ 1545 | 21020413 |
| 134 | ZSS | SD | Shandong | 114.36~118.26 | 34.22~38.23 | 0~ 1545 | 21020414 |
| 135 | ZSS | SD | Shandong | 114.36~118.26 | 34.22~38.23 | 0~ 1545 | 21020415 |
| 136 | ZSS | SD | Shandong | 114.36~118.26 | 34.22~38.23 | 0~ 1545 | 21020501 |
| 137 | ZSS | SD | Shandong | 114.36~118.26 | 34.22~38.23 | 0~ 1545 | 21020502 |
| 138 | ZSS | SD | Shandong | 114.36~118.26 | 34.22~38.23 | 0~ 1545 | 21020503 |
| 139 | ZSS | SD | Shandong | 114.36~118.26 | 34.22~38.23 | 0~ 1545 | 21020504 |
| 140 | ZSS | SD | Shandong | 114.36~118.26 | 34.22~38.23 | 0~ 1545 | 21020505 |
| 141 | ZSS | SD | Shandong | 114.36~118.26 | 34.22~38.23 | 0~ 1545 | 21020506 |
| 142 | ZSS | SD | Shandong | 114.36~118.26 | 34.22~38.23 | 0~ 1545 | 21020507 |
| 143 | ZSS | SD | Shandong | 114.36~118.26 | 34.22~38.23 | 0~ 1545 | 21020508 |
| 144 | ZSS | SD | Shandong | 114.36~118.26 | 34.22~38.23 | 0~ 1545 | 21020509 |
| 145 | ZSS | SD | Shandong | 114.36~118.26 | 34.22~38.23 | 0~ 1545 | 21020510 |
| 146 | ZSS | SD | Shandong | 114.36~118.26 | 34.22~38.23 | 0~ 1545 | 21020511 |
| 147 | ZSS | SD | Shandong | 114.36~118.26 | 34.22~38.23 | 0~ 1545 | 21020512 |
| 148 | ZSS | SD | Shandong | 114.36~118.26 | 34.22~38.23 | 0~ 1545 | 21020513 |
| 149 | ZSS | SD | Shandong | 114.36~118.26 | 34.22~38.23 | 0~ 1545 | 21020514 |
| 150 | ZSS | SD | Shandong | 114.36~118.26 | 34.22~38.23 | 0~ 1545 | 21020515 |
| 151 | ZSS | SN | Shaanxi | 105.29~111.15 | 31.42~39.35 | 169~3771 | 220707101 |
| 152 | ZSS | SN | Shaanxi | 105.29~111.15 | 31.42~39.35 | 169~3771 | 220707102 |
| 153 | ZSS | SN | Shaanxi | 105.29~111.15 | 31.42~39.35 | 169~3771 | 220707103 |
| 154 | ZSS | SN | Shaanxi | 105.29~111.15 | 31.42~39.35 | 169~3771 | 220707104 |
| 155 | ZSS | SN | Shaanxi | 105.29~111.15 | 31.42~39.35 | 169~3771 | 220707105 |
| 156 | ZSS | SN | Shaanxi | 105.29~111.15 | 31.42~39.35 | 169~3771 | 220707106 |
| 157 | ZSS | SN | Shaanxi | 105.29~111.15 | 31.42~39.35 | 169~3771 | 220707107 |
| 158 | ZSS | SN | Shaanxi | 105.29~111.15 | 31.42~39.35 | 169~3771 | 220707108 |
| 159 | ZSS | SN | Shaanxi | 105.29~111.15 | 31.42~39.35 | 169~3771 | 220707109 |
| 160 | ZSS | SN | Shaanxi | 105.29~111.15 | 31.42~39.35 | 169~3771 | 220707110 |
| 161 | ZSS | SN | Shaanxi | 105.29~111.15 | 31.42~39.35 | 169~3771 | 220707111 |
| 162 | ZSS | SN | Shaanxi | 105.29~111.15 | 31.42~39.35 | 169~3771 | 220707112 |
| 163 | ZSS | SN | Shaanxi | 105.29~111.15 | 31.42~39.35 | 169~3771 | 220707113 |
| 164 | ZSS | SN | Shaanxi | 105.29~111.15 | 31.42~39.35 | 169~3771 | 220707114 |
| 165 | ZSS | SN | Shaanxi | 105.29~111.15 | 31.42~39.35 | 169~3771 | 220707115 |
| 166 | ZSS | SN | Shaanxi | 105.29~111.15 | 31.42~39.35 | 169~3771 | 220707201 |
| 167 | ZSS | SN | Shaanxi | 105.29~111.15 | 31.42~39.35 | 169~3771 | 220707202 |
| 168 | ZSS | SN | Shaanxi | 105.29~111.15 | 31.42~39.35 | 169~3771 | 220707203 |
| 169 | ZSS | SN | Shaanxi | 105.29~111.15 | 31.42~39.35 | 169~3771 | 220707204 |
| 170 | ZSS | SN | Shaanxi | 105.29~111.15 | 31.42~39.35 | 169~3771 | 220707205 |
| 171 | ZSS | SN | Shaanxi | 105.29~111.15 | 31.42~39.35 | 169~3771 | 220707206 |
| 172 | ZSS | SN | Shaanxi | 105.29~111.15 | 31.42~39.35 | 169~3771 | 220707207 |
| 173 | ZSS | SN | Shaanxi | 105.29~111.15 | 31.42~39.35 | 169~3771 | 220707208 |
| 174 | ZSS | SN | Shaanxi | 105.29~111.15 | 31.42~39.35 | 169~3771 | 220707209 |
| 175 | ZSS | SN | Shaanxi | 105.29~111.15 | 31.42~39.35 | 169~3771 | 220707210 |
| 176 | ZSS | SN | Shaanxi | 105.29~111.15 | 31.42~39.35 | 169~3771 | 220707211 |
| 177 | ZSS | SN | Shaanxi | 105.29~111.15 | 31.42~39.35 | 169~3771 | 220707212 |
| 178 | ZSS | SN | Shaanxi | 105.29~111.15 | 31.42~39.35 | 169~3771 | 220707213 |
| 179 | ZSS | SN | Shaanxi | 105.29~111.15 | 31.42~39.35 | 169~3771 | 220707214 |
| 180 | ZSS | SN | Shaanxi | 105.29~111.15 | 31.42~39.35 | 169~3771 | 220707215 |
| 181 | ZSS | SN | Shaanxi | 105.29~111.15 | 31.42~39.35 | 169~3771 | 220707301 |
| 182 | ZSS | SN | Shaanxi | 105.29~111.15 | 31.42~39.35 | 169~3771 | 220707302 |
| 183 | ZSS | SN | Shaanxi | 105.29~111.15 | 31.42~39.35 | 169~3771 | 220707303 |
| 184 | ZSS | SN | Shaanxi | 105.29~111.15 | 31.42~39.35 | 169~3771 | 220707304 |
| 185 | ZSS | SN | Shaanxi | 105.29~111.15 | 31.42~39.35 | 169~3771 | 220707305 |
| 186 | ZSS | SN | Shaanxi | 105.29~111.15 | 31.42~39.35 | 169~3771 | 220707306 |
| 187 | ZSS | SN | Shaanxi | 105.29~111.15 | 31.42~39.35 | 169~3771 | 220707307 |
| 188 | ZSS | SN | Shaanxi | 105.29~111.15 | 31.42~39.35 | 169~3771 | 220707308 |
| 189 | ZSS | SN | Shaanxi | 105.29~111.15 | 31.42~39.35 | 169~3771 | 220707309 |
| 190 | ZSS | SN | Shaanxi | 105.29~111.15 | 31.42~39.35 | 169~3771 | 220707310 |
| 191 | ZSS | SN | Shaanxi | 105.29~111.15 | 31.42~39.35 | 169~3771 | 220707311 |
| 192 | ZSS | SN | Shaanxi | 105.29~111.15 | 31.42~39.35 | 169~3771 | 220707312 |
| 193 | ZSS | SN | Shaanxi | 105.29~111.15 | 31.42~39.35 | 169~3771 | 220707313 |
| 194 | ZSS | SN | Shaanxi | 105.29~111.15 | 31.42~39.35 | 169~3771 | 220707314 |
| 195 | ZSS | SN | Shaanxi | 105.29~111.15 | 31.42~39.35 | 169~3771 | 220707315 |
| 196 | ZSS | SN | Shaanxi | 105.29~111.15 | 31.42~39.35 | 169~3771 | 220707401 |
| 197 | ZSS | SN | Shaanxi | 105.29~111.15 | 31.42~39.35 | 169~3771 | 220707402 |
| 198 | ZSS | SN | Shaanxi | 105.29~111.15 | 31.42~39.35 | 169~3771 | 220707403 |
| 199 | ZSS | SN | Shaanxi | 105.29~111.15 | 31.42~39.35 | 169~3771 | 220707404 |
| 200 | ZSS | SN | Shaanxi | 105.29~111.15 | 31.42~39.35 | 169~3771 | 220707405 |
| 201 | ZSS | SN | Shaanxi | 105.29~111.15 | 31.42~39.35 | 169~3771 | 220707406 |
| 202 | ZSS | SN | Shaanxi | 105.29~111.15 | 31.42~39.35 | 169~3771 | 220707407 |
| 203 | ZSS | SN | Shaanxi | 105.29~111.15 | 31.42~39.35 | 169~3771 | 220707408 |
| 204 | ZSS | SN | Shaanxi | 105.29~111.15 | 31.42~39.35 | 169~3771 | 220707409 |
| 205 | ZSS | SN | Shaanxi | 105.29~111.15 | 31.42~39.35 | 169~3771 | 220707410 |
| 206 | ZSS | SN | Shaanxi | 105.29~111.15 | 31.42~39.35 | 169~3771 | 220707411 |
| 207 | ZSS | SN | Shaanxi | 105.29~111.15 | 31.42~39.35 | 169~3771 | 220707412 |
| 208 | ZSS | SN | Shaanxi | 105.29~111.15 | 31.42~39.35 | 169~3771 | 220707413 |
| 209 | ZSS | SN | Shaanxi | 105.29~111.15 | 31.42~39.35 | 169~3771 | 220707414 |
| 210 | ZSS | SN | Shaanxi | 105.29~111.15 | 31.42~39.35 | 169~3771 | 220707415 |
| 211 | ZSS | SN | Shaanxi | 105.29~111.15 | 31.42~39.35 | 169~3771 | 220707501 |
| 212 | ZSS | SN | Shaanxi | 105.29~111.15 | 31.42~39.35 | 169~3771 | 220707502 |
| 213 | ZSS | SN | Shaanxi | 105.29~111.15 | 31.42~39.35 | 169~3771 | 220707503 |
| 214 | ZSS | SN | Shaanxi | 105.29~111.15 | 31.42~39.35 | 169~3771 | 220707504 |
| 215 | ZSS | SN | Shaanxi | 105.29~111.15 | 31.42~39.35 | 169~3771 | 220707505 |
| 216 | ZSS | SN | Shaanxi | 105.29~111.15 | 31.42~39.35 | 169~3771 | 220707506 |
| 217 | ZSS | SN | Shaanxi | 105.29~111.15 | 31.42~39.35 | 169~3771 | 220707507 |
| 218 | ZSS | SN | Shaanxi | 105.29~111.15 | 31.42~39.35 | 169~3771 | 220707508 |
| 219 | ZSS | SN | Shaanxi | 105.29~111.15 | 31.42~39.35 | 169~3771 | 220707509 |
| 220 | ZSS | SN | Shaanxi | 105.29~111.15 | 31.42~39.35 | 169~3771 | 220707510 |
| 221 | ZSS | SN | Shaanxi | 105.29~111.15 | 31.42~39.35 | 169~3771 | 220707511 |
| 222 | ZSS | SN | Shaanxi | 105.29~111.15 | 31.42~39.35 | 169~3771 | 220707512 |
| 223 | ZSS | SN | Shaanxi | 105.29~111.15 | 31.42~39.35 | 169~3771 | 220707513 |
| 224 | ZSS | SN | Shaanxi | 105.29~111.15 | 31.42~39.35 | 169~3771 | 220707514 |
| 225 | ZSS | SN | Shaanxi | 105.29~111.15 | 31.42~39.35 | 169~3771 | 220707515 |
| 226 | ZSS | SX | Shanxi | 110.15~114.35 | 34.34~40.47 | 285~3061 | 220522201 |
| 227 | ZSS | SX | Shanxi | 110.15~114.35 | 34.34~40.47 | 285~3061 | 220522202 |
| 228 | ZSS | SX | Shanxi | 110.15~114.35 | 34.34~40.47 | 285~3061 | 220522203 |
| 229 | ZSS | SX | Shanxi | 110.15~114.35 | 34.34~40.47 | 285~3061 | 220522204 |
| 230 | ZSS | SX | Shanxi | 110.15~114.35 | 34.34~40.47 | 285~3061 | 220522205 |
| 231 | ZSS | SX | Shanxi | 110.15~114.35 | 34.34~40.47 | 285~3061 | 220522206 |
| 232 | ZSS | SX | Shanxi | 110.15~114.35 | 34.34~40.47 | 285~3061 | 220522207 |
| 233 | ZSS | SX | Shanxi | 110.15~114.35 | 34.34~40.47 | 285~3061 | 220522208 |
| 234 | ZSS | SX | Shanxi | 110.15~114.35 | 34.34~40.47 | 285~3061 | 220522209 |
| 235 | ZSS | SX | Shanxi | 110.15~114.35 | 34.34~40.47 | 285~3061 | 220522210 |
| 236 | ZSS | SX | Shanxi | 110.15~114.35 | 34.34~40.47 | 285~3061 | 220522211 |
| 237 | ZSS | SX | Shanxi | 110.15~114.35 | 34.34~40.47 | 285~3061 | 220522212 |
| 238 | ZSS | SX | Shanxi | 110.15~114.35 | 34.34~40.47 | 285~3061 | 220522213 |
| 239 | ZSS | SX | Shanxi | 110.15~114.35 | 34.34~40.47 | 285~3061 | 220522214 |
| 240 | ZSS | SX | Shanxi | 110.15~114.35 | 34.34~40.47 | 285~3061 | 220522215 |
| 241 | ZSS | SX | Shanxi | 110.15~114.35 | 34.34~40.47 | 285~3061 | 220522301 |
| 242 | ZSS | SX | Shanxi | 110.15~114.35 | 34.34~40.47 | 285~3061 | 220522302 |
| 243 | ZSS | SX | Shanxi | 110.15~114.35 | 34.34~40.47 | 285~3061 | 220522303 |
| 244 | ZSS | SX | Shanxi | 110.15~114.35 | 34.34~40.47 | 285~3061 | 220522304 |
| 245 | ZSS | SX | Shanxi | 110.15~114.35 | 34.34~40.47 | 285~3061 | 220522305 |
| 246 | ZSS | SX | Shanxi | 110.15~114.35 | 34.34~40.47 | 285~3061 | 220522306 |
| 247 | ZSS | SX | Shanxi | 110.15~114.35 | 34.34~40.47 | 285~3061 | 220522307 |
| 248 | ZSS | SX | Shanxi | 110.15~114.35 | 34.34~40.47 | 285~3061 | 220522308 |
| 249 | ZSS | SX | Shanxi | 110.15~114.35 | 34.34~40.47 | 285~3061 | 220522309 |
| 250 | ZSS | SX | Shanxi | 110.15~114.35 | 34.34~40.47 | 285~3061 | 220522310 |
| 251 | ZSS | SX | Shanxi | 110.15~114.35 | 34.34~40.47 | 285~3061 | 220522311 |
| 252 | ZSS | SX | Shanxi | 110.15~114.35 | 34.34~40.47 | 285~3061 | 220522312 |
| 253 | ZSS | SX | Shanxi | 110.15~114.35 | 34.34~40.47 | 285~3061 | 220522313 |
| 254 | ZSS | SX | Shanxi | 110.15~114.35 | 34.34~40.47 | 285~3061 | 220522314 |
| 255 | ZSS | SX | Shanxi | 110.15~114.35 | 34.34~40.47 | 285~3061 | 220522315 |
| 256 | ZSS | SX | Shanxi | 110.15~114.35 | 34.34~40.47 | 285~3061 | 220522401 |
| 257 | ZSS | SX | Shanxi | 110.15~114.35 | 34.34~40.47 | 285~3061 | 220522402 |
| 258 | ZSS | SX | Shanxi | 110.15~114.35 | 34.34~40.47 | 285~3061 | 220522403 |
| 259 | ZSS | SX | Shanxi | 110.15~114.35 | 34.34~40.47 | 285~3061 | 220522404 |
| 260 | ZSS | SX | Shanxi | 110.15~114.35 | 34.34~40.47 | 285~3061 | 220522405 |
| 261 | ZSS | SX | Shanxi | 110.15~114.35 | 34.34~40.47 | 285~3061 | 220522406 |
| 262 | ZSS | SX | Shanxi | 110.15~114.35 | 34.34~40.47 | 285~3061 | 220522407 |
| 263 | ZSS | SX | Shanxi | 110.15~114.35 | 34.34~40.47 | 285~3061 | 220522408 |
| 264 | ZSS | SX | Shanxi | 110.15~114.35 | 34.34~40.47 | 285~3061 | 220522409 |
| 265 | ZSS | SX | Shanxi | 110.15~114.35 | 34.34~40.47 | 285~3061 | 220522410 |
| 266 | ZSS | SX | Shanxi | 110.15~114.35 | 34.34~40.47 | 285~3061 | 220522411 |
| 267 | ZSS | SX | Shanxi | 110.15~114.35 | 34.34~40.47 | 285~3061 | 220522412 |
| 268 | ZSS | SX | Shanxi | 110.15~114.35 | 34.34~40.47 | 285~3061 | 220522413 |
| 269 | ZSS | SX | Shanxi | 110.15~114.35 | 34.34~40.47 | 285~3061 | 220522414 |
| 270 | ZSS | SX | Shanxi | 110.15~114.35 | 34.34~40.47 | 285~3061 | 220522415 |
| 271 | ZSS | SX | Shanxi | 110.15~114.35 | 34.34~40.47 | 285~3061 | 220522501 |
| 272 | ZSS | SX | Shanxi | 110.15~114.35 | 34.34~40.47 | 285~3061 | 220522502 |
| 273 | ZSS | SX | Shanxi | 110.15~114.35 | 34.34~40.47 | 285~3061 | 220522503 |
| 274 | ZSS | SX | Shanxi | 110.15~114.35 | 34.34~40.47 | 285~3061 | 220522504 |
| 275 | ZSS | SX | Shanxi | 110.15~114.35 | 34.34~40.47 | 285~3061 | 220522505 |
| 276 | ZSS | SX | Shanxi | 110.15~114.35 | 34.34~40.47 | 285~3061 | 220522506 |
| 277 | ZSS | SX | Shanxi | 110.15~114.35 | 34.34~40.47 | 285~3061 | 220522507 |
| 278 | ZSS | SX | Shanxi | 110.15~114.35 | 34.34~40.47 | 285~3061 | 220522508 |
| 279 | ZSS | SX | Shanxi | 110.15~114.35 | 34.34~40.47 | 285~3061 | 220522509 |
| 280 | ZSS | SX | Shanxi | 110.15~114.35 | 34.34~40.47 | 285~3061 | 220522510 |
| 281 | ZSS | SX | Shanxi | 110.15~114.35 | 34.34~40.47 | 285~3061 | 220522511 |
| 282 | ZSS | SX | Shanxi | 110.15~114.35 | 34.34~40.47 | 285~3061 | 220522512 |
| 283 | ZSS | SX | Shanxi | 110.15~114.35 | 34.34~40.47 | 285~3061 | 220522513 |
| 284 | ZSS | SX | Shanxi | 110.15~114.35 | 34.34~40.47 | 285~3061 | 220522514 |
| 285 | ZSS | SX | Shanxi | 110.15~114.35 | 34.34~40.47 | 285~3061 | 220522515 |
| 286 | ZSS | SX | Shanxi | 110.15~114.35 | 34.34~40.47 | 285~3061 | 220522601 |
| 287 | ZSS | SX | Shanxi | 110.15~114.35 | 34.34~40.47 | 285~3061 | 220522602 |
| 288 | ZSS | SX | Shanxi | 110.15~114.35 | 34.34~40.47 | 285~3061 | 220522603 |
| 289 | ZSS | SX | Shanxi | 110.15~114.35 | 34.34~40.47 | 285~3061 | 220522604 |
| 290 | ZSS | SX | Shanxi | 110.15~114.35 | 34.34~40.47 | 285~3061 | 220522605 |
| 291 | ZSS | SX | Shanxi | 110.15~114.35 | 34.34~40.47 | 285~3061 | 220522606 |
| 292 | ZSS | SX | Shanxi | 110.15~114.35 | 34.34~40.47 | 285~3061 | 220522607 |
| 293 | ZSS | SX | Shanxi | 110.15~114.35 | 34.34~40.47 | 285~3061 | 220522608 |
| 294 | ZSS | SX | Shanxi | 110.15~114.35 | 34.34~40.47 | 285~3061 | 220522609 |
| 295 | ZSS | SX | Shanxi | 110.15~114.35 | 34.34~40.47 | 285~3061 | 220522610 |
| 296 | ZSS | SX | Shanxi | 110.15~114.35 | 34.34~40.47 | 285~3061 | 220522611 |
| 297 | ZSS | SX | Shanxi | 110.15~114.35 | 34.34~40.47 | 285~3061 | 220522612 |
| 298 | ZSS | SX | Shanxi | 110.15~114.35 | 34.34~40.47 | 285~3061 | 220522613 |
| 299 | ZSS | SX | Shanxi | 110.15~114.35 | 34.34~40.47 | 285~3061 | 220522614 |
| 300 | ZSS | SX | Shanxi | 110.15~114.35 | 34.34~40.47 | 285~3061 | 220522615 |
| 301 | ZMS | YN | Yunnan | 97.31~106.11 | 21.08~29.15 | 76.4~6740 | 202201031 |
| 302 | ZMS | YN | Yunnan | 97.31~106.11 | 21.08~29.15 | 76.4~6740 | 202201032 |
| 303 | ZMS | YN | Yunnan | 97.31~106.11 | 21.08~29.15 | 76.4~6740 | 202201033 |
| 304 | ZMS | YN | Yunnan | 97.31~106.11 | 21.08~29.15 | 76.4~6740 | 202201034 |
| 305 | ZMS | YN | Yunnan | 97.31~106.11 | 21.08~29.15 | 76.4~6740 | 202201035 |
| 306 | ZMS | YN | Yunnan | 97.31~106.11 | 21.08~29.15 | 76.4~6740 | 202201036 |
| 307 | ZMS | YN | Yunnan | 97.31~106.11 | 21.08~29.15 | 76.4~6740 | 202201037 |
| 308 | ZMS | YN | Yunnan | 97.31~106.11 | 21.08~29.15 | 76.4~6740 | 202201038 |
| 309 | ZMS | YN | Yunnan | 97.31~106.11 | 21.08~29.15 | 76.4~6740 | 202201039 |
| 310 | ZMS | YN | Yunnan | 97.31~106.11 | 21.08~29.15 | 76.4~6740 | 202201040 |
| 311 | ZMS | YN | Yunnan | 97.31~106.11 | 21.08~29.15 | 76.4~6740 | 202202011 |
| 312 | ZMS | YN | Yunnan | 97.31~106.11 | 21.08~29.15 | 76.4~6740 | 202202012 |
| 313 | ZMS | YN | Yunnan | 97.31~106.11 | 21.08~29.15 | 76.4~6740 | 202202013 |
| 314 | ZMS | YN | Yunnan | 97.31~106.11 | 21.08~29.15 | 76.4~6740 | 202202014 |
| 315 | ZMS | YN | Yunnan | 97.31~106.11 | 21.08~29.15 | 76.4~6740 | 202202015 |
| 316 | ZMS | YN | Yunnan | 97.31~106.11 | 21.08~29.15 | 76.4~6740 | 202202016 |
| 317 | ZMS | YN | Yunnan | 97.31~106.11 | 21.08~29.15 | 76.4~6740 | 202202017 |
| 318 | ZMS | YN | Yunnan | 97.31~106.11 | 21.08~29.15 | 76.4~6740 | 202202018 |
| 319 | ZMS | YN | Yunnan | 97.31~106.11 | 21.08~29.15 | 76.4~6740 | 202202019 |
| 320 | ZMS | YN | Yunnan | 97.31~106.11 | 21.08~29.15 | 76.4~6740 | 202202020 |
| 321 | ZMS | YN | Yunnan | 97.31~106.11 | 21.08~29.15 | 76.4~6740 | 202202171 |
| 322 | ZMS | YN | Yunnan | 97.31~106.11 | 21.08~29.15 | 76.4~6740 | 202202172 |
| 323 | ZMS | YN | Yunnan | 97.31~106.11 | 21.08~29.15 | 76.4~6740 | 202202173 |
| 324 | ZMS | YN | Yunnan | 97.31~106.11 | 21.08~29.15 | 76.4~6740 | 202202174 |
| 325 | ZMS | YN | Yunnan | 97.31~106.11 | 21.08~29.15 | 76.4~6740 | 202202175 |
| 326 | ZMS | YN | Yunnan | 97.31~106.11 | 21.08~29.15 | 76.4~6740 | 202202176 |
| 327 | ZMS | YN | Yunnan | 97.31~106.11 | 21.08~29.15 | 76.4~6740 | 202202177 |
| 328 | ZMS | YN | Yunnan | 97.31~106.11 | 21.08~29.15 | 76.4~6740 | 202202178 |
| 329 | ZMS | YN | Yunnan | 97.31~106.11 | 21.08~29.15 | 76.4~6740 | 202202179 |
| 330 | ZMS | YN | Yunnan | 97.31~106.11 | 21.08~29.15 | 76.4~6740 | 202202180 |
| 331 | ZMS | YN | Yunnan | 97.31~106.11 | 21.08~29.15 | 76.4~6740 | 202202181 |
| 332 | ZMS | YN | Yunnan | 97.31~106.11 | 21.08~29.15 | 76.4~6740 | 202202182 |
| 333 | ZMS | YN | Yunnan | 97.31~106.11 | 21.08~29.15 | 76.4~6740 | 202202183 |
| 334 | ZMS | YN | Yunnan | 97.31~106.11 | 21.08~29.15 | 76.4~6740 | 202202184 |
| 335 | ZMS | YN | Yunnan | 97.31~106.11 | 21.08~29.15 | 76.4~6740 | 202202185 |
| 336 | ZMS | YN | Yunnan | 97.31~106.11 | 21.08~29.15 | 76.4~6740 | 202202186 |
| 337 | ZMS | YN | Yunnan | 97.31~106.11 | 21.08~29.15 | 76.4~6740 | 202202187 |
| 338 | ZMS | YN | Yunnan | 97.31~106.11 | 21.08~29.15 | 76.4~6740 | 202202188 |
| 339 | ZMS | YN | Yunnan | 97.31~106.11 | 21.08~29.15 | 76.4~6740 | 202202189 |
| 340 | ZMS | YN | Yunnan | 97.31~106.11 | 21.08~29.15 | 76.4~6740 | 202202190 |
| 341 | ZMS | YN | Yunnan | 97.31~106.11 | 21.08~29.15 | 76.4~6740 | 202203221 |
| 342 | ZMS | YN | Yunnan | 97.31~106.11 | 21.08~29.15 | 76.4~6740 | 202203222 |
| 343 | ZMS | YN | Yunnan | 97.31~106.11 | 21.08~29.15 | 76.4~6740 | 202203223 |
| 344 | ZMS | YN | Yunnan | 97.31~106.11 | 21.08~29.15 | 76.4~6740 | 202203224 |
| 345 | ZMS | YN | Yunnan | 97.31~106.11 | 21.08~29.15 | 76.4~6740 | 202203225 |
| 346 | ZMS | YN | Yunnan | 97.31~106.11 | 21.08~29.15 | 76.4~6740 | 202203226 |
| 347 | ZMS | YN | Yunnan | 97.31~106.11 | 21.08~29.15 | 76.4~6740 | 202203227 |
| 348 | ZMS | YN | Yunnan | 97.31~106.11 | 21.08~29.15 | 76.4~6740 | 202203228 |
| 349 | ZMS | YN | Yunnan | 97.31~106.11 | 21.08~29.15 | 76.4~6740 | 202203229 |
| 350 | ZMS | YN | Yunnan | 97.31~106.11 | 21.08~29.15 | 76.4~6740 | 202203230 |
| 351 | ZMS | YN | Yunnan | 97.31~106.11 | 21.08~29.15 | 76.4~6740 | 202203411 |
| 352 | ZMS | YN | Yunnan | 97.31~106.11 | 21.08~29.15 | 76.4~6740 | 202203412 |
| 353 | ZMS | YN | Yunnan | 97.31~106.11 | 21.08~29.15 | 76.4~6740 | 202203413 |
| 354 | ZMS | YN | Yunnan | 97.31~106.11 | 21.08~29.15 | 76.4~6740 | 202203414 |
| 355 | ZMS | YN | Yunnan | 97.31~106.11 | 21.08~29.15 | 76.4~6740 | 202203415 |
| 356 | ZMS | YN | Yunnan | 97.31~106.11 | 21.08~29.15 | 76.4~6740 | 202203416 |
| 357 | ZMS | YN | Yunnan | 97.31~106.11 | 21.08~29.15 | 76.4~6740 | 202203417 |
| 358 | ZMS | YN | Yunnan | 97.31~106.11 | 21.08~29.15 | 76.4~6740 | 202203418 |
| 359 | ZMS | YN | Yunnan | 97.31~106.11 | 21.08~29.15 | 76.4~6740 | 202203419 |
| 360 | ZMS | YN | Yunnan | 97.31~106.11 | 21.08~29.15 | 76.4~6740 | 202203420 |
| 361 | ZMS | YN | Yunnan | 97.31~106.11 | 21.08~29.15 | 76.4~6740 | 202203441 |
| 362 | ZMS | YN | Yunnan | 97.31~106.11 | 21.08~29.15 | 76.4~6740 | 202203442 |
| 363 | ZMS | YN | Yunnan | 97.31~106.11 | 21.08~29.15 | 76.4~6740 | 202203443 |
| 364 | ZMS | YN | Yunnan | 97.31~106.11 | 21.08~29.15 | 76.4~6740 | 202203444 |
| 365 | ZMS | YN | Yunnan | 97.31~106.11 | 21.08~29.15 | 76.4~6740 | 202203445 |
| 366 | ZMS | YN | Yunnan | 97.31~106.11 | 21.08~29.15 | 76.4~6740 | 202203446 |
| 367 | ZMS | YN | Yunnan | 97.31~106.11 | 21.08~29.15 | 76.4~6740 | 202203447 |
| 368 | ZMS | YN | Yunnan | 97.31~106.11 | 21.08~29.15 | 76.4~6740 | 202203448 |
| 369 | ZMS | YN | Yunnan | 97.31~106.11 | 21.08~29.15 | 76.4~6740 | 202203449 |
| 370 | ZMS | YN | Yunnan | 97.31~106.11 | 21.08~29.15 | 76.4~6740 | 202203450 |
| 371 | ZMS | YN | Yunnan | 97.31~106.11 | 21.08~29.15 | 76.4~6740 | 202204291 |
| 372 | ZMS | YN | Yunnan | 97.31~106.11 | 21.08~29.15 | 76.4~6740 | 202204292 |
| 373 | ZMS | YN | Yunnan | 97.31~106.11 | 21.08~29.15 | 76.4~6740 | 202204293 |
| 374 | ZMS | YN | Yunnan | 97.31~106.11 | 21.08~29.15 | 76.4~6740 | 202204294 |
| 375 | ZMS | YN | Yunnan | 97.31~106.11 | 21.08~29.15 | 76.4~6740 | 202204295 |
| 376 | ZMS | YN | Yunnan | 97.31~106.11 | 21.08~29.15 | 76.4~6740 | 202204296 |
| 377 | ZMS | YN | Yunnan | 97.31~106.11 | 21.08~29.15 | 76.4~6740 | 202204297 |
| 378 | ZMS | YN | Yunnan | 97.31~106.11 | 21.08~29.15 | 76.4~6740 | 202204298 |
| 379 | ZMS | YN | Yunnan | 97.31~106.11 | 21.08~29.15 | 76.4~6740 | 202204299 |
| 380 | ZMS | YN | Yunnan | 97.31~106.11 | 21.08~29.15 | 76.4~6740 | 202204300 |
| 381 | ZMS | YN | Yunnan | 97.31~106.11 | 21.08~29.15 | 76.4~6740 | 202207101 |
| 382 | ZMS | YN | Yunnan | 97.31~106.11 | 21.08~29.15 | 76.4~6740 | 202207102 |
| 383 | ZMS | YN | Yunnan | 97.31~106.11 | 21.08~29.15 | 76.4~6740 | 202207103 |
| 384 | ZMS | YN | Yunnan | 97.31~106.11 | 21.08~29.15 | 76.4~6740 | 202207104 |
| 385 | ZMS | YN | Yunnan | 97.31~106.11 | 21.08~29.15 | 76.4~6740 | 202207105 |
| 386 | ZMS | YN | Yunnan | 97.31~106.11 | 21.08~29.15 | 76.4~6740 | 202207106 |
| 387 | ZMS | YN | Yunnan | 97.31~106.11 | 21.08~29.15 | 76.4~6740 | 202207107 |
| 388 | ZMS | YN | Yunnan | 97.31~106.11 | 21.08~29.15 | 76.4~6740 | 202207108 |
| 389 | HAS | AH | Anhui | 114.24~119.37 | 29.41~34.38 | 21.8~1864.8 | 21213101 |
| 390 | HAS | AH | Anhui | 114.24~119.37 | 29.41~34.38 | 21.8~1864.8 | 21213102 |
| 391 | HAS | AH | Anhui | 114.24~119.37 | 29.41~34.38 | 21.8~1864.8 | 21213103 |
| 392 | HAS | AH | Anhui | 114.24~119.37 | 29.41~34.38 | 21.8~1864.8 | 21213104 |
| 393 | HAS | AH | Anhui | 114.24~119.37 | 29.41~34.38 | 21.8~1864.8 | 21213105 |
| 394 | HAS | AH | Anhui | 114.24~119.37 | 29.41~34.38 | 21.8~1864.8 | 21213106 |
| 395 | HAS | AH | Anhui | 114.24~119.37 | 29.41~34.38 | 21.8~1864.8 | 21213107 |
| 396 | HAS | AH | Anhui | 114.24~119.37 | 29.41~34.38 | 21.8~1864.8 | 21213108 |
| 397 | HAS | AH | Anhui | 114.24~119.37 | 29.41~34.38 | 21.8~1864.8 | 21213109 |
| 398 | HAS | AH | Anhui | 114.24~119.37 | 29.41~34.38 | 21.8~1864.8 | 21213110 |
| 399 | HAS | AH | Anhui | 114.24~119.37 | 29.41~34.38 | 21.8~1864.8 | 21213201 |
| 400 | HAS | AH | Anhui | 114.24~119.37 | 29.41~34.38 | 21.8~1864.8 | 21213202 |
| 401 | HAS | AH | Anhui | 114.24~119.37 | 29.41~34.38 | 21.8~1864.8 | 21213203 |
| 402 | HAS | AH | Anhui | 114.24~119.37 | 29.41~34.38 | 21.8~1864.8 | 21213204 |
| 403 | HAS | AH | Anhui | 114.24~119.37 | 29.41~34.38 | 21.8~1864.8 | 21213205 |
| 404 | HAS | AH | Anhui | 114.24~119.37 | 29.41~34.38 | 21.8~1864.8 | 21213206 |
| 405 | HAS | AH | Anhui | 114.24~119.37 | 29.41~34.38 | 21.8~1864.8 | 21213207 |
| 406 | HAS | AH | Anhui | 114.24~119.37 | 29.41~34.38 | 21.8~1864.8 | 21213208 |
| 407 | HAS | AH | Anhui | 114.24~119.37 | 29.41~34.38 | 21.8~1864.8 | 21213209 |
| 408 | HAS | AH | Anhui | 114.24~119.37 | 29.41~34.38 | 21.8~1864.8 | 21213210 |
| 409 | HAS | AH | Anhui | 114.24~119.37 | 29.41~34.38 | 21.8~1864.8 | 21213301 |
| 410 | HAS | AH | Anhui | 114.24~119.37 | 29.41~34.38 | 21.8~1864.8 | 21213302 |
| 411 | HAS | AH | Anhui | 114.24~119.37 | 29.41~34.38 | 21.8~1864.8 | 21213303 |
| 412 | HAS | AH | Anhui | 114.24~119.37 | 29.41~34.38 | 21.8~1864.8 | 21213304 |
| 413 | HAS | AH | Anhui | 114.24~119.37 | 29.41~34.38 | 21.8~1864.8 | 21213305 |
| 414 | HAS | AH | Anhui | 114.24~119.37 | 29.41~34.38 | 21.8~1864.8 | 21213306 |
| 415 | HAS | AH | Anhui | 114.24~119.37 | 29.41~34.38 | 21.8~1864.8 | 21213307 |
| 416 | HAS | AH | Anhui | 114.24~119.37 | 29.41~34.38 | 21.8~1864.8 | 21213308 |
| 417 | HAS | AH | Anhui | 114.24~119.37 | 29.41~34.38 | 21.8~1864.8 | 21213309 |
| 418 | HAS | AH | Anhui | 114.24~119.37 | 29.41~34.38 | 21.8~1864.8 | 21213310 |
| 419 | HAS | AH | Anhui | 114.24~119.37 | 29.41~34.38 | 21.8~1864.8 | 21213401 |
| 420 | HAS | AH | Anhui | 114.24~119.37 | 29.41~34.38 | 21.8~1864.8 | 21213402 |
| 421 | HAS | AH | Anhui | 114.24~119.37 | 29.41~34.38 | 21.8~1864.8 | 21213403 |
| 422 | HAS | AH | Anhui | 114.24~119.37 | 29.41~34.38 | 21.8~1864.8 | 21213404 |
| 423 | HAS | AH | Anhui | 114.24~119.37 | 29.41~34.38 | 21.8~1864.8 | 21213405 |
| 424 | HAS | AH | Anhui | 114.24~119.37 | 29.41~34.38 | 21.8~1864.8 | 21213406 |
| 425 | HAS | AH | Anhui | 114.24~119.37 | 29.41~34.38 | 21.8~1864.8 | 21213407 |
| 426 | HAS | AH | Anhui | 114.24~119.37 | 29.41~34.38 | 21.8~1864.8 | 21213408 |
| 427 | HAS | AH | Anhui | 114.24~119.37 | 29.41~34.38 | 21.8~1864.8 | 21213409 |
| 428 | HAS | AH | Anhui | 114.24~119.37 | 29.41~34.38 | 21.8~1864.8 | 21213410 |
| 429 | HAS | AH | Anhui | 114.24~119.37 | 29.41~34.38 | 21.8~1864.8 | 21213501 |
| 430 | HAS | AH | Anhui | 114.24~119.37 | 29.41~34.38 | 21.8~1864.8 | 21213502 |
| 431 | HAS | AH | Anhui | 114.24~119.37 | 29.41~34.38 | 21.8~1864.8 | 21213503 |
| 432 | HAS | AH | Anhui | 114.24~119.37 | 29.41~34.38 | 21.8~1864.8 | 21213504 |
| 433 | HAS | AH | Anhui | 114.24~119.37 | 29.41~34.38 | 21.8~1864.8 | 21213505 |
| 434 | HAS | AH | Anhui | 114.24~119.37 | 29.41~34.38 | 21.8~1864.8 | 21213506 |
| 435 | HAS | AH | Anhui | 114.24~119.37 | 29.41~34.38 | 21.8~1864.8 | 21213507 |
| 436 | HAS | AH | Anhui | 114.24~119.37 | 29.41~34.38 | 21.8~1864.8 | 21213508 |
| 437 | HAS | AH | Anhui | 114.24~119.37 | 29.41~34.38 | 21.8~1864.8 | 21213509 |
| 438 | HAS | AH | Anhui | 114.24~119.37 | 29.41~34.38 | 21.8~1864.8 | 21213510 |
| 439 | HAS | AH | Anhui | 114.24~119.37 | 29.41~34.38 | 21.8~1864.8 | 21213601 |
| 440 | HAS | AH | Anhui | 114.24~119.37 | 29.41~34.38 | 21.8~1864.8 | 21213602 |
| 441 | HAS | AH | Anhui | 114.24~119.37 | 29.41~34.38 | 21.8~1864.8 | 21213603 |
| 442 | HAS | AH | Anhui | 114.24~119.37 | 29.41~34.38 | 21.8~1864.8 | 21213604 |
| 443 | HAS | AH | Anhui | 114.24~119.37 | 29.41~34.38 | 21.8~1864.8 | 21213605 |
| 444 | HAS | AH | Anhui | 114.24~119.37 | 29.41~34.38 | 21.8~1864.8 | 21213606 |
| 445 | HAS | AH | Anhui | 114.24~119.37 | 29.41~34.38 | 21.8~1864.8 | 21213607 |
| 446 | HAS | AH | Anhui | 114.24~119.37 | 29.41~34.38 | 21.8~1864.8 | 21213608 |
| 447 | HAS | AH | Anhui | 114.24~119.37 | 29.41~34.38 | 21.8~1864.8 | 21213609 |
| 448 | HAS | AH | Anhui | 114.24~119.37 | 29.41~34.38 | 21.8~1864.8 | 21213610 |
| 449 | HAS | AH | Anhui | 114.24~119.37 | 29.41~34.38 | 21.8~1864.8 | 21213701 |
| 450 | HAS | AH | Anhui | 114.24~119.37 | 29.41~34.38 | 21.8~1864.8 | 21213702 |
| 451 | HAS | AH | Anhui | 114.24~119.37 | 29.41~34.38 | 21.8~1864.8 | 21213703 |
| 452 | HAS | AH | Anhui | 114.24~119.37 | 29.41~34.38 | 21.8~1864.8 | 21213704 |
| 453 | HAS | AH | Anhui | 114.24~119.37 | 29.41~34.38 | 21.8~1864.8 | 21213705 |
| 454 | HAS | AH | Anhui | 114.24~119.37 | 29.41~34.38 | 21.8~1864.8 | 21213706 |
| 455 | HAS | AH | Anhui | 114.24~119.37 | 29.41~34.38 | 21.8~1864.8 | 21213707 |
| 456 | HAS | AH | Anhui | 114.24~119.37 | 29.41~34.38 | 21.8~1864.8 | 21213708 |
| 457 | HAS | AH | Anhui | 114.24~119.37 | 29.41~34.38 | 21.8~1864.8 | 21213709 |
| 458 | HAS | AH | Anhui | 114.24~119.37 | 29.41~34.38 | 21.8~1864.8 | 21213710 |
| 459 | HAS | AH | Anhui | 114.24~119.37 | 29.41~34.38 | 21.8~1864.8 | 21321101 |
| 460 | HAS | AH | Anhui | 114.24~119.37 | 29.41~34.38 | 21.8~1864.8 | 21321102 |
| 461 | HAS | AH | Anhui | 114.24~119.37 | 29.41~34.38 | 21.8~1864.8 | 21321103 |
| 462 | HAS | AH | Anhui | 114.24~119.37 | 29.41~34.38 | 21.8~1864.8 | 21321104 |
| 463 | HAS | AH | Anhui | 114.24~119.37 | 29.41~34.38 | 21.8~1864.8 | 21321105 |
| 464 | HAS | AH | Anhui | 114.24~119.37 | 29.41~34.38 | 21.8~1864.8 | 21321106 |
| 465 | HAS | AH | Anhui | 114.24~119.37 | 29.41~34.38 | 21.8~1864.8 | 21321107 |
| 466 | HAS | AH | Anhui | 114.24~119.37 | 29.41~34.38 | 21.8~1864.8 | 21321108 |
| 467 | HAS | AH | Anhui | 114.24~119.37 | 29.41~34.38 | 21.8~1864.8 | 21321109 |
| 468 | HAS | AH | Anhui | 114.24~119.37 | 29.41~34.38 | 21.8~1864.8 | 21321110 |
| 469 | HAS | AH | Anhui | 114.24~119.37 | 29.41~34.38 | 21.8~1864.8 | 21321201 |
| 470 | HAS | AH | Anhui | 114.24~119.37 | 29.41~34.38 | 21.8~1864.8 | 21321202 |
| 471 | HAS | AH | Anhui | 114.24~119.37 | 29.41~34.38 | 21.8~1864.8 | 21321203 |
| 472 | HAS | AH | Anhui | 114.24~119.37 | 29.41~34.38 | 21.8~1864.8 | 21321204 |
| 473 | HAS | AH | Anhui | 114.24~119.37 | 29.41~34.38 | 21.8~1864.8 | 21321205 |
| 474 | HAS | AH | Anhui | 114.24~119.37 | 29.41~34.38 | 21.8~1864.8 | 21321206 |
| 475 | HAS | AH | Anhui | 114.24~119.37 | 29.41~34.38 | 21.8~1864.8 | 21321207 |
| 476 | HAS | AH | Anhui | 114.24~119.37 | 29.41~34.38 | 21.8~1864.8 | 21321208 |
| 477 | HAS | AH | Anhui | 114.24~119.37 | 29.41~34.38 | 21.8~1864.8 | 21321209 |
| 478 | HAS | AH | Anhui | 114.24~119.37 | 29.41~34.38 | 21.8~1864.8 | 21321210 |
| 479 | HAS | AH | Anhui | 114.24~119.37 | 29.41~34.38 | 21.8~1864.8 | 21321301 |
| 480 | HAS | AH | Anhui | 114.24~119.37 | 29.41~34.38 | 21.8~1864.8 | 21321302 |
| 481 | HAS | AH | Anhui | 114.24~119.37 | 29.41~34.38 | 21.8~1864.8 | 21321303 |
| 482 | HAS | AH | Anhui | 114.24~119.37 | 29.41~34.38 | 21.8~1864.8 | 21321304 |
| 483 | HAS | AH | Anhui | 114.24~119.37 | 29.41~34.38 | 21.8~1864.8 | 21321305 |
| 484 | HAS | AH | Anhui | 114.24~119.37 | 29.41~34.38 | 21.8~1864.8 | 21321306 |
| 485 | HAS | AH | Anhui | 114.24~119.37 | 29.41~34.38 | 21.8~1864.8 | 21321307 |
| 486 | HAS | AH | Anhui | 114.24~119.37 | 29.41~34.38 | 21.8~1864.8 | 21321308 |
| 487 | HAS | AH | Anhui | 114.24~119.37 | 29.41~34.38 | 21.8~1864.8 | 21321309 |
| 488 | HAS | AH | Anhui | 114.24~119.37 | 29.41~34.38 | 21.8~1864.8 | 21321310 |

**Table. S2** Fire ion dimensionality reduction algorithm based on chromaticity value feature extraction.

import os
import cv2
import numpy as np
import pandas as pd

# Function to extract Lab features
def extract_lab_features(image):
 lab_image = cv2.cvtColor(image, cv2.COLOR_BGR2LAB)
 l_channel = lab_image[:, :, 0]
 a_channel = lab_image[:, :, 1]
 b_channel = lab_image[:, :, 2]

 lab_mean = np.mean(lab_image, axis=(0, 1))
 lab_std = np.std(lab_image, axis=(0, 1))

 lab_features = np.concatenate((lab_mean, lab_std), axis=None)

 return lab_features

# Function to extract RGB features
def extract_rgb_features(image):
 r_channel = image[:, :, 2]
 g_channel = image[:, :, 1]
 b_channel = image[:, :, 0]

 rgb_mean = np.mean(image, axis=(0, 1))
 rgb_std = np.std(image, axis=(0, 1))

 rgb_features = np.concatenate((rgb_mean, rgb_std), axis=None)

 return rgb_features

# Function to extract HSI features
def extract_hsi_features(image):
 hsi_image = cv2.cvtColor(image, cv2.COLOR_BGR2HSV)
 h_channel = hsi_image[:, :, 0]
 s_channel = hsi_image[:, :, 1]
 i_channel = hsi_image[:, :, 2]

 hsi_mean = np.mean(hsi_image, axis=(0, 1))
 hsi_std = np.std(hsi_image, axis=(0, 1))

 hsi_features = np.concatenate((hsi_mean, hsi_std), axis=None)

 return hsi_features

# Algorithm for extracting fire foreground
def extract_fire_foreground(image):
 lab_image = cv2.cvtColor(image, cv2.COLOR_BGR2LAB)
 l_channel = lab_image[:, :, 0]
 a_channel = lab_image[:, :, 1]
 b_channel = lab_image[:, :, 2]

 r_channel = image[:, :, 2]
 g_channel = image[:, :, 1]
 b_channel = image[:, :, 0]

 hsi_image = cv2.cvtColor(image, cv2.COLOR_BGR2HSV)
 h_channel = hsi_image[:, :, 0]
 s_channel = hsi_image[:, :, 1]
 i_channel = hsi_image[:, :, 2]

 _, otsu_thresh = cv2.threshold(cv2.cvtColor(image, cv2.COLOR_BGR2GRAY), 0, 255, cv2.THRESH_BINARY + cv2.THRESH_OTSU)

 fire_foreground = np.zeros_like(otsu_thresh)
 fire_foreground[(l_channel > 50) & (a_channel > 128) & (b_channel > 128) & (r_channel > 150) & (g_channel > 150) & (b_channel < 100) & (h_channel > 0) & (h_channel < 30) & (s_channel > 50) & (i_channel > 100)] = 255
 fire_foreground[otsu_thresh > 0] = 255

 return fire_foreground

# Fire detection function
def fire_detection(image):
 fire_foreground = extract_fire_foreground(image)
 lab_features = extract_lab_features(image)
 rgb_features = extract_rgb_features(image)
 hsi_features = extract_hsi_features(image)

 return fire_foreground, lab_features, rgb_features, hsi_features

# Process all images in a folder and save results to Excel
def process_folder_and_save_to_excel(folder_path, output_excel):
 results = []

 for filename in os.listdir(folder_path):
 if filename.endswith('.jpg') or filename.endswith('.png'):
 image_path = os.path.join(folder_path, filename)
 image = cv2.imread(image_path)

 fire_foreground, lab_features, rgb_features, hsi_features = fire_detection(image)

 result = {
 'Image': filename,
 'Result': fire_foreground.mean(),
 'Lab_Mean': lab_features[:3],
 'Lab_Std': lab_features[3:],
 'RGB_Mean': rgb_features[:3],
 'RGB_Std': rgb_features[3:],
 'HSI_Mean': hsi_features[:3],
 'HSI_Std': hsi_features[3:]
 }
 results.append(result)

 save_folder = 'fire'
 if not os.path.exists(save_folder):
 os.makedirs(save_folder)

 cv2.imwrite(os.path.join(save_folder, filename), fire_foreground)

 df = pd.DataFrame(results)
 df.to_excel(output_excel, index=False)

# Set the image folder path and output Excel file path
image_folder = "a"
output_excel_path = "fire_detection_results.xlsx"

# Process images in the folder and save results to Excel
process_folder_and_save_to_excel(image_folder, output_excel_path)

print("Results have been saved to the Excel file.")

**Table. S3** Ice ion dimensionality reduction algorithm based on texture feature extraction.

import cv2
import numpy as np
import pandas as pd
import os
import matplotlib.pyplot as plt
from skimage.feature import greycomatrix, greycoprops


def calculate_glcm(image):
 # Calculate GLCM features for a grayscale image
 glcm = greycomatrix(image, distances=[1], angles=[0], levels=256, symmetric=True, normed=True)
 contrast = greycoprops(glcm, 'contrast')[0, 0]
 dissimilarity = greycoprops(glcm, 'dissimilarity')[0, 0]
 homogeneity = greycoprops(glcm, 'homogeneity')[0, 0]
 energy = greycoprops(glcm, 'energy')[0, 0]
 correlation = greycoprops(glcm, 'correlation')[0, 0]
 return np.array([contrast, dissimilarity, homogeneity, energy, correlation])


def apply_laws_kernels(image):
 # Define Laws' texture energy kernels
 kernels = [
 np.array([1, 4, 6, 4, 1]), # L5 (Level)
 np.array([-1, -2, 0, 2, 1]), # E5 (Edge)
 np.array([-1, 0, 2, 0, -1]), # S5 (Spot)
 np.array([-1, 2, 0, -2, 1]), # R5 (Ripple)
 np.array([1, -4, 6, -4, 1]) # D5 (Difference)
 ]

 laws_energy_features = []

 for kernel in kernels:
 kernel = kernel[:, np.newaxis] @ kernel[np.newaxis, :]
 filtered_image = cv2.filter2D(image, -1, kernel)
 energy = np.sum(filtered_image ** 2)
 laws_energy_features.append(energy)

 return np.array(laws_energy_features)


def extract_ice_foreground(image):
 gray_image = cv2.cvtColor(image, cv2.COLOR_BGR2GRAY)

 # Calculate GLCM features
 glcm_features = calculate_glcm(gray_image)

 # Calculate Laws' texture energy features
 laws_features = apply_laws_kernels(gray_image)

 # Combine features
 ice_foreground = np.hstack((glcm_features, laws_features))
 return ice_foreground


def ice_detection(image):
 ice_foreground = extract_ice_foreground(image)
 return ice_foreground


def process_folder_and_save_to_excel(folder_path, output_excel):
 results = []

 for filename in os.listdir(folder_path):
 if filename.endswith('.jpg') or filename.endswith('.png'):
 image_path = os.path.join(folder_path, filename)
 image = cv2.imread(image_path)

 ice_foreground = ice_detection(image)

 result = {
 'Image': filename,
 'Result': np.mean(ice_foreground)
 }
 results.append(result)

 plt.figure()
 plt.subplot(1, 2, 1)
 plt.imshow(cv2.cvtColor(image, cv2.COLOR_BGR2RGB))
 plt.title('Original Image')
 plt.axis('off')

 plt.subplot(1, 2, 2)
 plt.plot(ice_foreground, marker='o', linestyle='none')
 plt.title('Ice Detection Features')
 plt.axis('off')

 plt.show()

 df = pd.DataFrame(results)
 df.to_excel(output_excel, index=False)


width = 500
height = 1100
folder_path = 'yw'
images_data = []

for file_name in os.listdir(folder_path):
 if file_name.endswith('.png') or file_name.endswith('.jpg'):
 image = cv2.imread(os.path.join(folder_path, file_name))
 image_resized = cv2.resize(image, (width, height))
 images_data.append(image_resized)

images_data = np.array(images_data)

output_excel_path = "ice_detection_results.xlsx"
process_folder_and_save_to_excel(folder_path, output_excel_path)

print("Results have been saved to the Excel file.")


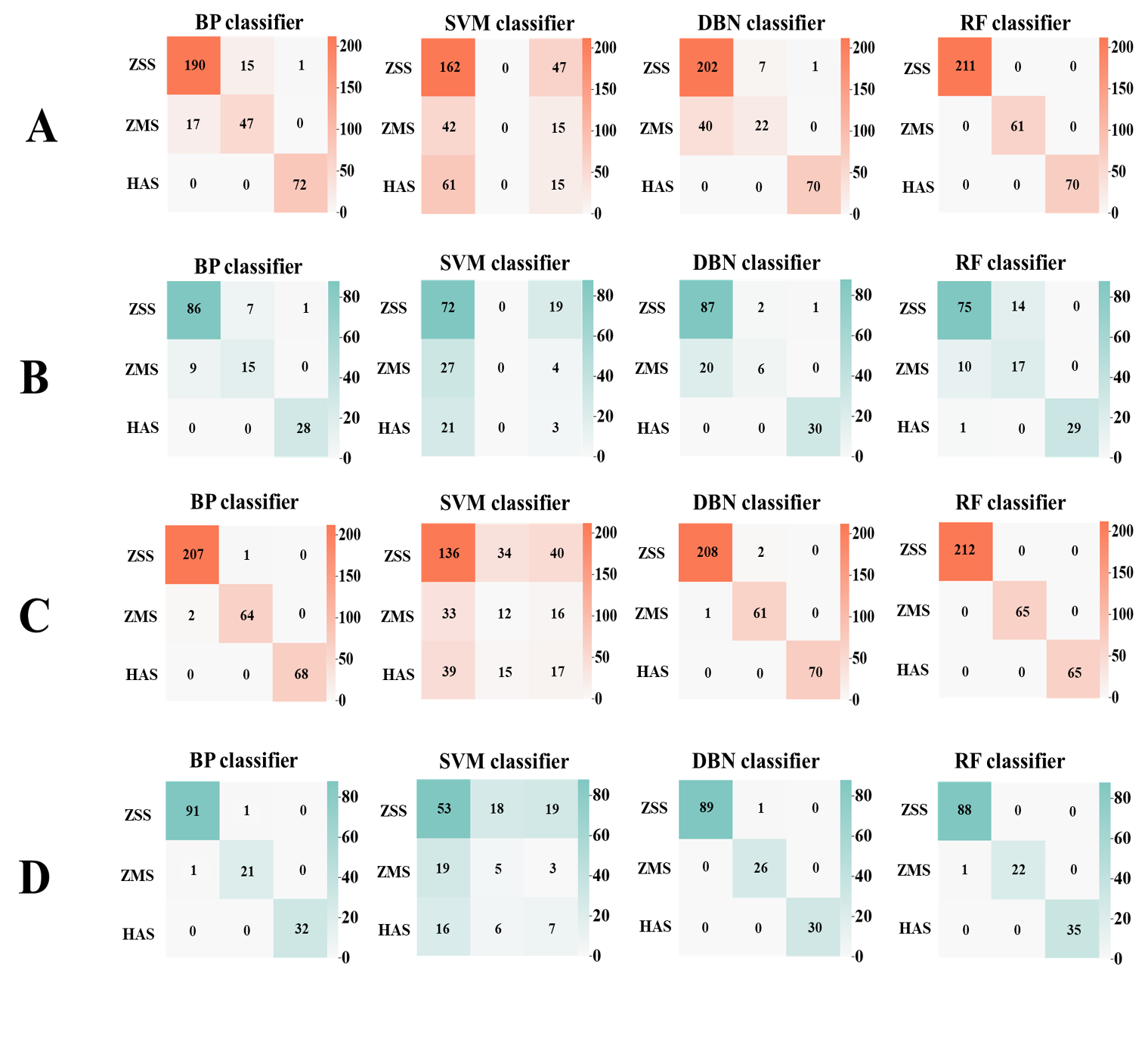


**Figure S1.** Confusion matrices of the train set (A) and test set (B) of 4 machine learning algorithms based on the original data for classification discrimination; Confusion matrices of the training set (C) and test set (D) of 4 machine learning algorithms based on the fire-ice ion data for classification discrimination.

ZSS, Ziziphi Spinosae Semen; ZMS, Ziziphi Mauritianae Semen; HAS, Hovenia Acerba Semen.
